# Cardiac Organoid Model Inspired Micro‐Robot Smart Patch to Treat Myocardial Infarction

**DOI:** 10.1002/adma.202417327

**Published:** 2025-03-06

**Authors:** Fangfang Wang, Zilu Xu, Feiyang Zheng, Dongcheng Yang, Kaige Xu, Ming Ke, Xiao Zhou, Xiaohan Zhang, Ju Tan, Yong Liu, Yansha Hao, Malcolm Xing, Chuhong Zhu

**Affiliations:** ^1^ Department of Anatomy Engineering Research Center for Organ Intelligent Biological Manufacturing of Chongqing Key Lab for Biomechanics and Tissue Engineering of Chongqing Third Military Medical University Chongqing 400038 China; ^2^ Engineering Research Center of Ministry of Education for Tissue and Organ Regeneration and Manufacturing Chongqing 400038 China; ^3^ School of Medicine & Nursing, and First Affiliated Hospital Huzhou University Huzhou 313000 China; ^4^ Department of Mechanical Engineering University of Manitoba Winnipeg R3T 2N2 Canada; ^5^ National Key Laboratory of Trauma and Chemical Poisoning Chongqing 400038 China

**Keywords:** asymmetric micro‐robot in micro‐needle, exosome, hypoxic‐acidic microenvironment, human cardiac organoid, myocardium infarction

## Abstract

Post‐myocardial infarction (MI), the rapid decrease in pH triggers myocardial cell acidosis, which compromises the therapeutic efficacy of exosomes in MI. The groundbreaking research in the human cardiac organoid MI model suggests that exosomes, when paired with pH adjustment, dramatically reduce cardiomyocyte mortality while maintaining their proliferative potential, underscoring the importance of pH regulation in myocardial preservation. Micro‐robot mounted micro‐needle (MN) patch is thus proposed, targeting MI‐acidic microenvironmet, to deliver exosomes into deep injured tissue. Upon injection, the patch base releases VEGF‐laden nanoparticles adhering to the infarcted myocardium. The smart patch is found not only 3D reconstructs the vascular network in MI regions but also effectively saves cardiomyocytes in rats. Furthermore, the minimally invasive delivery of MN patches are also verified to hearts of rabbits and pigs via thoracoscopic surgery underscores. These findings suggest that precise regulation of the microenvironment is a key to improving treatment outcomes.

## Graphical abstract

The heart organoid model exhibits the acidic microenvironment characteristic of myocardial infarction, which emerges as a pivotal force propelling the movement of micro‐robots. These micro‐robots, administered through microneedles, can penetrate deep into the tissue, effectively delivering therapeutic payloads to facilitate heart repair.

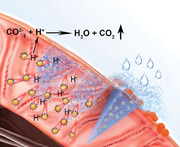

## 1 Introduction

Post‐myocardial infarction (MI), blood flow to the affected area is obstructed, leading to a swift increase in local carbon dioxide concentration. External pH rapidly drops from 7.4 to as low as 5.9,^[^ ^1^ ^]^ thereby acting on various ion channels. Most of the ion channels that contribute to cardiac action potentials, such as K^+[^ ^1b^ ^]^ channels and Ca^2+[^ ^2^ ^]^ channels, are inhibited by extracellular acidosis. In particular, the inhibition of slow calcium currents directly results in a decrease in myocardial contractility.^[^ ^3^ ^]^ At the same time, the accumulation of hydrogen ions (H^+^) and lactate ions in cells also leads to a decrease in the intracellular pH (pH_i_).^[^ ^4^ ^]^ Early in ischemia, the development of acidosis may serve as a regulatory mechanism to decrease contractility and ATP consumption, and thus to preserve cellular integrity; however, in later stages of ischemia, a more severe acidosis may contribute to cell necrosis.^[^ ^5^ ^]^ Cardiomyocyte survive and myocardial contractility seem to be closely related to pH levels, but the importance of pH might be often overlooked.

Well‐directed evidence suggests that exosomes derived from mesenchymal stem cells excel in cardiac repair through stimulating cardiomyocyte proliferation,^[^ ^6^ ^]^ vascular angiogenesis,^[^ ^7^ ^]^ immune regulation,^[^ ^8^ ^]^ and inhibiting the progression of scar formation.^[^ ^9^ ^]^ Nonetheless, exosomes administered via intravenous injection are easily trapped in non‐targeted organs, especially in the lungs and liver,^[^ ^10^ ^]^ resulting in insufficient targeting of the myocardial ischemia area, making it difficult to reach the infarct area. Even with intracardiac injection, it is challenging to ensure the retention and penetration of exosomes within the infarcted areas. Moreover, the slightly acidic environment in the infarct regions poses a significant threat to the survival of myocardial cells, which undoubtedly greatly compromises the therapeutic efficacy of exosomes.

Utilizing the human cardiac organoid (HCO) to circumvent the above limitations, we verified that in the hypoxic‐acidic environment, increasing the pH of the acidic medium could enhance the exosomes' ability to regulate anaerobic metabolism in cardiomyocytes.

Self‐driven bio‐robots in micro and nano, a “maxime miranda in minimis”, are considered to be next‐generation drug delivery solutions fit for the micro‐environment.^[^ ^11^ ^]^ These bio‐robots, upon different stimuli such as enzyme,^[^ ^12^ ^]^ pH,^[^ ^13^ ^]^ light,^[^ ^14^ ^]^ and electric‐magnetics^[^ ^15^ ^]^ (**Table** **1**), can heap all the profits that other vehicles are unable to. The fact that the pH drops to ≈5.5 when MI happens^[^ ^16^ ^]^ inspires us to conceive self‐driven asymmetric microparticles from egg‐shell‐membrane. The CaCO_3_ nature of the eggshell on one side and the acid‐resistant protein membrane on the other side led us to consider the design of a microrobot with directional movement in a weak, acidic MI scene.^[^ ^13^ ^]^ The membrane has a second motive to provide a bioorthogonal platform to link exomes with metabolite cleavable bonds. The micro‐needle (MN) can overcome the current limitations of the cardiac patches only cast onto the surface, which cannot fully integrate and repair infarcted tissue.^[^ ^17^ ^]^ With the needle's penetration, the therapeutic drugs are accurately and sustainably released in the infarcted area, ensuring that the myocardial tissue receives effective treatment from the outside in. Added one further benefit, we consider the designed MNs to be flexible, foldable, and operatable via video‐assisted thoracoscopic surgery on‐demand transported to the myocardium. The innovative integration of MN patches and self‐driven bio‐robots will undoubtedly bring new hope for MI treatment.

**Table 1:** Micro‐robots with Different Power Sources.

Materials	Size	Power sources	Applications
Mesoporous silica nanoparticles containing urease enzymes and gold nanoparticles	Average diameter of 507.8 ± 3.4 nm	Urease enzyme	Drug targeting and delivering^[^ ^12^ ^]^
Carbonate and tranexamic acid	Average diameter of 10 mm	Gas‐generating	Delivering therapeutics into wounds^[^ ^13^ ^]^
Liquid crystalline elastomer	14.8 mm long, 3.8 mm wide, and 50 µm thick	Spatially modulated light field	Walking up a slope, squeezing through a narrow slit, and pushing objects^[^ ^14^ ^]^
Photocurable gelatin–methacryloyl (GelMA)‐based hydrogel composite multiferroic nanoparticles	About 100 µm long	Low magnitude rotating magnetic fields	Neuronal cell delivering, in situ neuronal stimulating and biodegrading/ Inducing the differentiation of neuronal cells^[^ ^15a^ ^]^
Porous magnetic cobalt–nickel alloy	Rectangular micropillars (length: 100 µm; width: 30 µm; height: 30 µm)	Customized magnetic actuation system	Highly efficient, targeted drug‐delivery system^[^ ^15b^ ^]^

**Figure 1:**
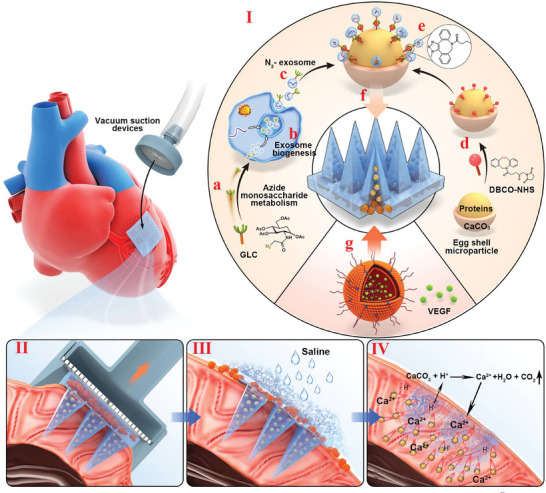
Schematic showing the overall study design of the secondary drug‐loaded microneedle (MN) patch. I)MN patch construction: a–c) harvesting azide‐modified exosomes (N_3_‐exosomes) through mesenchymal stem cells metabolizing azide monosaccharides Ac4GlcNAz (Glc); d‐e) N_3_‐exosomes linked to DBCO‐NHS modified eggshell microparticles (ESMP) through a bioorthogonal reaction; f) the exosome‐to‐eggshell complex encapsulated into needle tips of the MN patch; and g) the vascular endothelial growth factor (VEGF)‐encapsulating dual complementary liposome (DLC‐VEGF) nanoparticles incorporated into the effervescent base of MN patches; then II) the patch administered into the ischemic heart with the assistance of a customized apparatus; III) the DLC‐VEGF nanoparticles burst‐released into myocardial infarction (MI) regions through the effervescent base rapidly dissolving; and IV) exosomes delivered to deeper and wider MI regions by self‐propelled ESMPs.

Our device features a two‐stage drug delivery from MN patch: 1) VEGF in dual complementary liposome nanoparticles (DLC‐VEGF) in patch base, mainly used for early vascular network reconstruction,^[^ ^18^ ^]^ 2) self‐driven micro‐robots in needles aiming to enhance the local pH and deliver exosomes for avoiding adverse VR.^[^ ^19^ ^]^ **Figure** **1** illustrates the assembly process of self‐driven MN patches to treat MI. Human mesenchymal stem cells (HMSCs) metabolize the bioorthogonal target molecule, azide monosaccharide Ac4GlcNAz (Glc),^[^ ^20^ ^]^ to harvest azide‐modified exosomes (N_3_‐exosomes). N_3_‐exosomes are linked with the protein membrane side of eggshell microparticle, which is pre‐modified with dibenzocyclooctyne‐N‐hydroxysuccinimidyl ester (DBCO‐NHS), through a bioorthogonal reaction.^[^ ^21^ ^]^ The asymmetric microrobots would response to MI‐acidic zone to carry exosomes to the distal regions upon fueled by the impetus. The synergy effect is unparalleled, as demonstrated in the following aspects: 1) the needle penetration plus powered particles to achieve such long‐distance transportation of exomes via micro‐robots to distal MI sites, 2) ESMPs plus exosomes regulating cardiomyocyte metabolism in hypoxic environment on the basis of enhancing the local pH, and 3) VEGF plus exosomes to achieve the 3D reconstruction of the vascular network at the infarction site.

## 2 Results

### 2.1 Constructing a Novel Combined Therapeutic Approach Targeting the Acidic Microenvironment for MI

**Figure 2:**
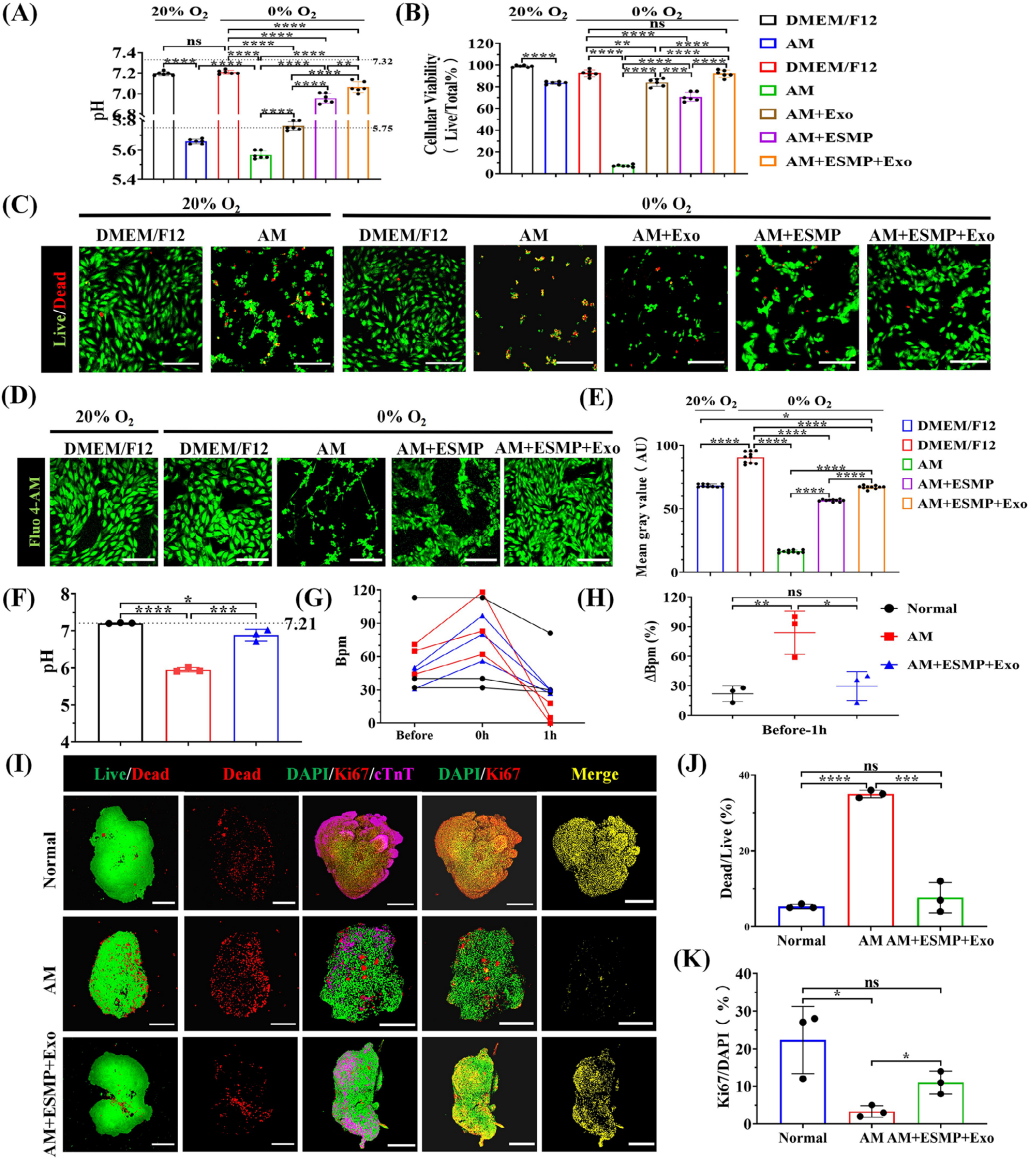
Human cardiac organoids verifying the therapeutic efficacy in targeting the hypoxic acidic microenvironment formed after MI. A) Comparative analysis of the pH of H9C2 cells after 2 h of acidic culture in the 20% or 0% O_2_ environment (*n* = 6). The pH of DMEM/F12 is 7.32 and the pH of AM is 5.75. B,C) Quantitative analysis of the survival rate of H9C2 cells after 2 h of hypoxic‐acidic culture using ImageJ (*n* = 6). Scale bar: 200 µm. D,E) Quantitative analysis of the intracellular calcium ion accumulation in H9C2 cells after 2 h of hypoxic‐acidic culture using ImageJ (*n* = 10). Scale bar: 200 µm. F) Comparative analysis of the pH of HCOs after 1‐h incubation in 0% O_2_ environment (*n* = 3). The pH of HCO maintenance medium is 7.21. G,H) Comparative analysis of the beating frequency of HCOs after 1 hour of hypoxic‐acidic culture (*n* = 3). I) Representative immunofluorescence images of HCOs in the control and treatment groups. Yellow fluorescent dots indicated the co‐localization area of DAPI (green) and Ki67 (red). Scale bar: 500 µm. J,K) Quantitative analysis of the cell viability percentage and Ki67 positive rate of HCOs using ImageJ (*n* = 3). All data were presented as means ± SD. Comparisons between two groups were performed using two‐tailed unpaired Student's t test, and statistical significance was indicated by asterisks (*) above the lines. NS indicates *p* > 0.1234. *p < 0.0332, and **p < 0.0021. ***p < 0.0002, and ****p < 0.0001.

We prepared an acidic medium (AM) by adding 2.4974 m glucose to physiological saline, adjusting the pH to 5.75. Cardiomyocytes were incubated in the acidic medium under conditions of 5% CO_2_ and 95% N_2_ (equivalent to 0% O_2_) to replicate the hypoxic‐acidic microenvironment that arises post‐MI. Dulbecco's Modified Eagle's Medium/F12 (DMEM/F12) supplemented with 10% fetal bovine serum was used as a positive control to assess the effects of acidic environment under anoxic conditions on cardiomyocytes. The results revealed that regardless of whether in an aerobic or anoxic environment, cells cultured in DMEM/F12 maintained their pH value with very limited variation, due to the inherent buffering capacity of the medium, and were highly consistent with the initial pH of the culture medium (7.210 ± 0.018 vs 7.197 ± 0.019 vs 7.32) (**Figure** **2**A). In the anaerobic environment of the AM group, the pH level measured was 5.567 ± 0.027, contrasting with the aerobic culture's 5.658 ± 0.016. This discrepancy might be attributed to the accumulation of lactic acid in anaerobic conditions. Nevertheless, their pH values were not substantially distinct from the AM's pH of 5.75. The exosome treatment group (Exo) maintained a consistent pH range of 5.762 ± 0.031. Notably, in the eggshell microparticle (ESMP) group and the ESMP combined with exosome treatment (ESMP + Exo) group, the pH experienced a significant rise to 6.957 ± 0.047 and 7.067 ± 0.053, respectively, with the latter showing slightly higher than the former. This alteration could be attributed to the neutralizing effect of calcium carbonate and the potential role of exosomes in metabolic regulation.

After 2 h of anaerobic culture, we performed live‐dead staining and conducted statistical analysis using ImageJ software (Figure 2B,C). Under 20% O_2_ culture conditions, the cell survival rate of the AM group was relatively high (83% ± 1.366%), but the cells exhibited obvious aggregation and shrinkage. In the AM group, subjected to hypoxic‐acidic conditions, the cell viability decreased to 7.333% ± 1.033%, and the cells essentially lost their intact morphology, existing as cell fragments. Although the live‐dead staining ratio of the Exo group was statistically superior to that of the ESMP group (84.17% ± 3.545% vs 70.83% ± 4.215%), the cellular condition of the former was notably worse than that of the latter. In the Exo group, cells generally showed significant shrinkage, while in the ESMP group, cells clustered but still maintained relatively intact cell morphology. It should be mentioned that the cell survival rate in the ESMP + Exo group showed no significant difference compared with the hypoxic DMEM/F12 group (92.17% ± 3.251% VS 92.50% ± 3.017%), and cells maintained a good morphology.

Subsequently, we conducted a statistical analysis of the intracellular calcium accumulation in cardiomyocytes that had undergone a 2‐h hypoxic treatment. Here, we used DMEM/F12 as a positive control and added an equal amount of 1.0507 m calcium chloride to the acidic medium. As the pH value of the solution remained essentially unchanged before and after the addition of calcium chloride, we decided to continue to label it as AM. As described in the literature, the results showed that the intracellular calcium ion gray value of the DMEM/F12 group under anoxic conditions was significantly higher than that under 20% oxygen conditions (90.43 ± 4.392 vs 68.28 ± 1.205) (Figure 2E). In the hypoxic‐acidic environment, the gray value of the AM group was the lowest, reaching only 16.50 ± 1.162. Although both the ESMP and the ESMP + Exo groups were added with eggshell microparticles, the gray value of the ESMP + Exo group was significantly higher than that of the ESMP group (66.96 ± 1.301 vs 56.69 ± 0.926), which might be attributed to the role of exosomes in metabolic regulation. Notably, the calcium ion accumulation in the ESMP + Exo group was much lower than that in the anoxic DMEM/F12 group and was similar to that in the aerobic DMEM/F12 group, which undoubtedly benefited the long‐term survival of the myocardial cells in the ESMP + Exo group. Cytomorphological observations further confirmed these results: the ESMP + Exo group showed regular cell morphology, while the cells in the AM group exhibited significant shrinkage, and the cells in the ESMP group demonstrated a significant trend of aggregation (Figure 2D). In conclusion, the combined application of calcium carbonate and exosomes not only takes advantage of carbonate ions to rapidly regulate pH, supplying calcium ions to maintain the ionic balance of cardiomyocytes, but also the comprehensive regulatory effect of exosomes further promotes the metabolic function of myocardial cells.

### 2.2 Therapeutic Efficacy Evaluation of Calcium Carbonate Combined with Exosomes for MI Using HCOs

To assess the potential efficacy of the combined treatment using calcium carbonate and exosomes for MI, HCOs were exposed to hypoxic‐acidic conditions, replicating the hypoxic and slightly acidic microenvironment that arises post‐MI. Since clinical MI is typically localized to specific regions of the heart, the size limitations of cardiac organoids make it difficult to achieve localized infarction, and the hypoxia‐acidic environment also pose a severe challenge to the survival of cardiomyocytes. Consequently, we conducted a preliminary experiment in the standard cell incubator (with 20% O_2_). The results revealed that the acidic significantly elevated the beating frequency of HCOs, yet this increased activity ceased after just 20 min (Figure S1, Supporting Information). Subsequently, cardiac organoids were subjected to hypoxic‐acidic environment for an hour and receiving treatment. The pH value remained steady at 7.21 ± 0.010 for the group using the standard cardiac organoid maintenance culture medium (Normal) (Figure 2F; Videos S1–S3, Supporting Information). Remarkably, the pH in the ESMP + Exo treatment group was substantially higher than that of the untreated group (AM), registering 6.88 ± 0.157 and 5.95 ± 0.061, respectively. Statistical analysis of the beating frequency demonstrated that the AM group experienced the most substantial relative change, reaching 84% ± 21.930% (Figure 2G,H). In contrast, the ESMP + Exo treatment group exhibited no significant variation when juxtaposed with the Normal group, posting relative changes of 29% ± 14.570% and 22% ± 7.937%, respectively. The beat amplitude of HCOs in both the ESMP + Exo group and the Normal group exhibited a reduction, which might be attributed to the heart's intrinsic self‐regulatory mechanisms in response to hypoxic conditions (Videos S1 and S3, Supporting Information).

We conducted an analysis on the viability and proliferation capacity of HCOs. In the Normal group, HCOs were maintained in an environment with 20% O_2_ using a conventional medium. The statistical analysis of live‐dead fluorescence staining revealed that the cell mortality rate of HCOs in the AM group was as high as 35% ± 1.000%, with a small number of small cell clumps formed by several cells; whereas the mortality rate in the ESMP + Exo treatment group was substantially lower, showing no statistically significant difference when compared with the Normal group (7.667% ± 4.041% VS 5.333% ± 0.577%) (Figure 2I,J). Regarding cell proliferation capacity, the Ki67 positive rate in the ESMP + Exo group was 11.00% ± 3.000%, which was not statistically significant different from the 12.33% ± 8.963% in the Normal group, but was significantly higher than the 3.33% ± 1.528% in the AM group (Figure 2I,K). These findings suggested that the combined application of calcium carbonate and exosomes harbors significant potential in the MI treatment.

### 2.3 Construction and Characterization of Azide‐Modified Exosomes (N_3_‐Exosomes)

**Figure 3:**
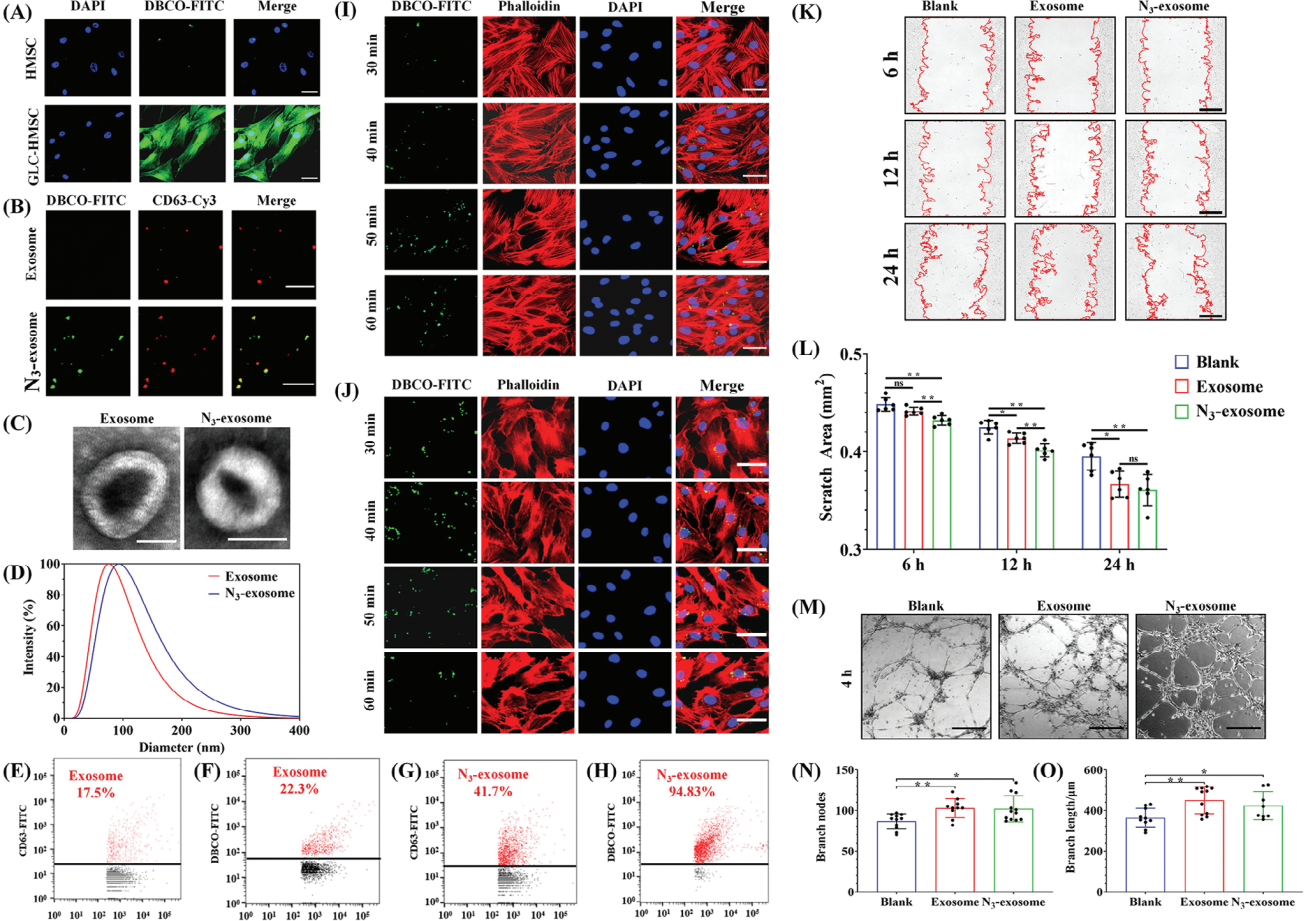
Characterization of N_3_‐exosomes. A) Fluorescent detection of Glc metabolism in HMSCs. DBCO‐FITC probes (green) specifically recognizing azide groups through a bioorthogonal reaction. Scale bar, 50 µm. B) Fluorescent staining to identify N_3_‐exosomes. CD63 (red) specifically labeled exosomes co‐localizing with azide groups (green). Scale bar, 10 µm. C) N_3_‐exosome identification via TEM. Scale bar, 100 µm. D) Measurement of N_3_‐exosome size using a particle size analyzer. E–H) Detection of N_3_‐exosomes by nanofluidic cytometry. I–J) Fluorescence images of H9C2 and HUVECs co‐incubated with N_3_‐exosomes. DBCO‐FITC‐labeled N_3_‐exosomes (green) merged with phalloidin (red)‐labeled cytoskeleton. Scale bar, 50 µm. K) Scratch migration assays of the control, exosome, and N_3_‐exosome groups. Scale bar, 200 µm. L) Quantification of scratch area (*n* = 6) by Image J. M) Representative images displaying tube formation of HUVECs in the control, exosome, and N_3_‐exosome groups. Scale bar, 250 µm. N,O) Quantification of branch nodes (*n* = 8) and branch length (*n* = 8) using Image J. All data were presented as means ± SD. Comparisons between two groups were performed using two‐tailed unpaired Student's t test, and statistical significance was indicated by asterisks (*) above the lines. NS indicates *p* > 0.1234. *p < 0.0332, and **p < 0.0021. ***p < 0.0002, and ****p < 0.0001.

Identification of HMSCs treated with azide monosaccharide Glc using a fluorescence microscope show sufficient metabolism of Glc in HMSCs (Glc‐HMSC) (**Figure** **3**A). Glc is nontoxic to HMSCs from cell proliferation assay (Figure S2, Supporting Information). Azide‐modified exosomes (N_3_‐exosomes) could be specifically labeled by DBCO‐FITC probes through a highly selective bioorthogonal reaction, but not in unmodified exosomes (Figure 3B). Cytometry further confirms that N3‐exosomes were labeled by both DBCO‐FITC probe and exosome marker CD63^[^ ^22^ ^]^ (Figure 3E–H). Thus, it suggests that azide groups were successfully grafted onto exosomes of Glc‐HMSCs. Transmission electron microscopy (TEM) images show that both N_3_‐exosomes and exosomes exhibit typical cup‐shaped morphology with an inward curvature (Figure 3C) with the size of ≈100 nm, consistent with reports^[^ ^23^ ^]^ (Figure 3D).

The internalization of N_3_‐exosomes by H9C2 (embryonic cardiomyocyte cell line) and HUVECs (human umbilical vein endothelial cells) was conducted, and we found that exosomes were internalized by cells within 30 min (Figure 3I,J). In addition, N_3_‐exosomes also promoted the growth of H9C2 and HUVECs (Figure S3A–D). After that, the effect of N_3_‐exosomes on endothelial cell functions was further studied. Quantification of the scratch assay revealed that both N_3_‐exosomes and unmodified exosomes accelerated the migration of endothelial cells, and there was no difference between them (Figure 3K,L). Similarly, there are also no difference in tube number or length during tube formation between azide‐exosomes and unmodified exosomes (Figure 3M–O). These results suggest that the modification did not change exosomes’ function.

### 2.4 Characterization of Vascular Endothelial Growth Factor (VEGF)‐Encapsulating dual Complementary Liposome (DLC‐VEGF)

**Figure 4:**
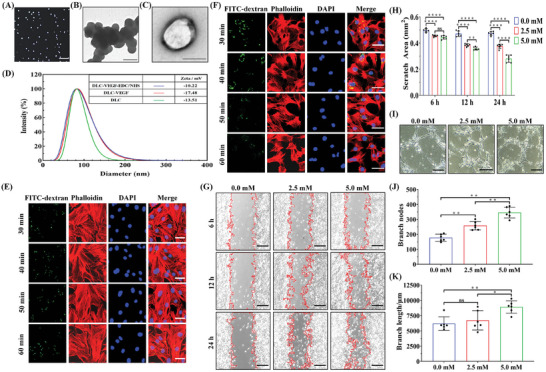
Characterization of DLC‐VEGF lipid particles. A) Microscopic visualization of DLC‐VEGF particles. Scale bar, 20 µm. B,C) TEM images of DLC‐VEGF particles. Scale bar, 200 nm. D) Measurement of DLC‐VEGF particle size and zeta potential. The particle size and zeta potential of DLC, DLC‐VEGF, and EDC/NHS modified DLC‐VEGF (DLC‐VEGF‐EDC/NHS) are analyzed. E,F) Fluorescence images of H9C2 and HUVECs incubated with DLC‐FITC particles. DLC‐FITC nanoparticles (green) co‐localized with phalloidin (red)‐labeled cytoskeleton. Scale bar, 50 µm. G,H) Assessment of the impact of DLC‐VEGF on HUVECs migration. The scratch area is quantified using ImageJ software (*n* = 6). Scale bar, 250 µm. I–K) Evaluation of the effect of DLC‐VEGF on HUVECs tube formation. Branch nodes and branch length are quantified through ImageJ (*n* = 6). Scale bar, 250 µm. Scale bar, 50 µm. All data are provided as means ± SD. Comparisons between two groups were performed using two‐tailed unpaired Student's t test, and statistical significance was indicated by the asterisks (*) above the lines. NS indicates *p* > 0.1234. *p < 0.0332, and **p < 0.0021, and ****p < 0.0001.

The dual complementary liposome (DLC) nanoparticles were prepared using a lipid extruder. The collected DLC‐VEGF nanoparticles were chemically modified with EDC/NHS, thereby obtaining the DLC‐VEGF‐EDC/NHS nanoparticles. Microscopic images show that the highly diluted lipid nanoparticles have a regular shape and relatively uniform particle size (**Figure** **4**A). The results of TEM reveal that the lipid particles are spherical with a diameter of ≈100 nm (Figure 4B,C). Furthermore, the diameters of DLC, DLC‐VEGF, and DLC‐VEGF‐EDC/NHS nanoparticles are all ≈100 nm (Figure 4D). And the changes in potentials indicate the successful encapsulation of VEGF in DLC nanoparticles and modification of EDC/NHS on DLC‐VEGF particles.

To test whether lipid particles can enter H9C2 and HUVECs, the particles encapsulating FITC‐dextran were prepared as a model. We found that DLC‐FITC nanoparticles were endocytosed by H9C2‐ and HUVECs within 30 min (Figure 4E,F). At the same time, DLC‐VEGF nanoparticles promote the proliferation of H9C2 and HUVECs (Figure S4A,B, Supporting Information). After ensuring the safe and effective internalization of DLC‐VEGF by HUVECs, the effect of DLC‐VEGF on endothelial cells was further verified. The scratch assay shows that compared with the control group, DLC‐VEGF effectively promotes the migration of endothelial cells and this tendency is even obvious when the concentration of DLC‐VEGF is raised (Figure 4G,H). In response to this, tube formation number and length are also increased (Figure 4I–K).

### 2.5 Characterization of Eggshell Microparticles (ESMP)

**Figure 5:**
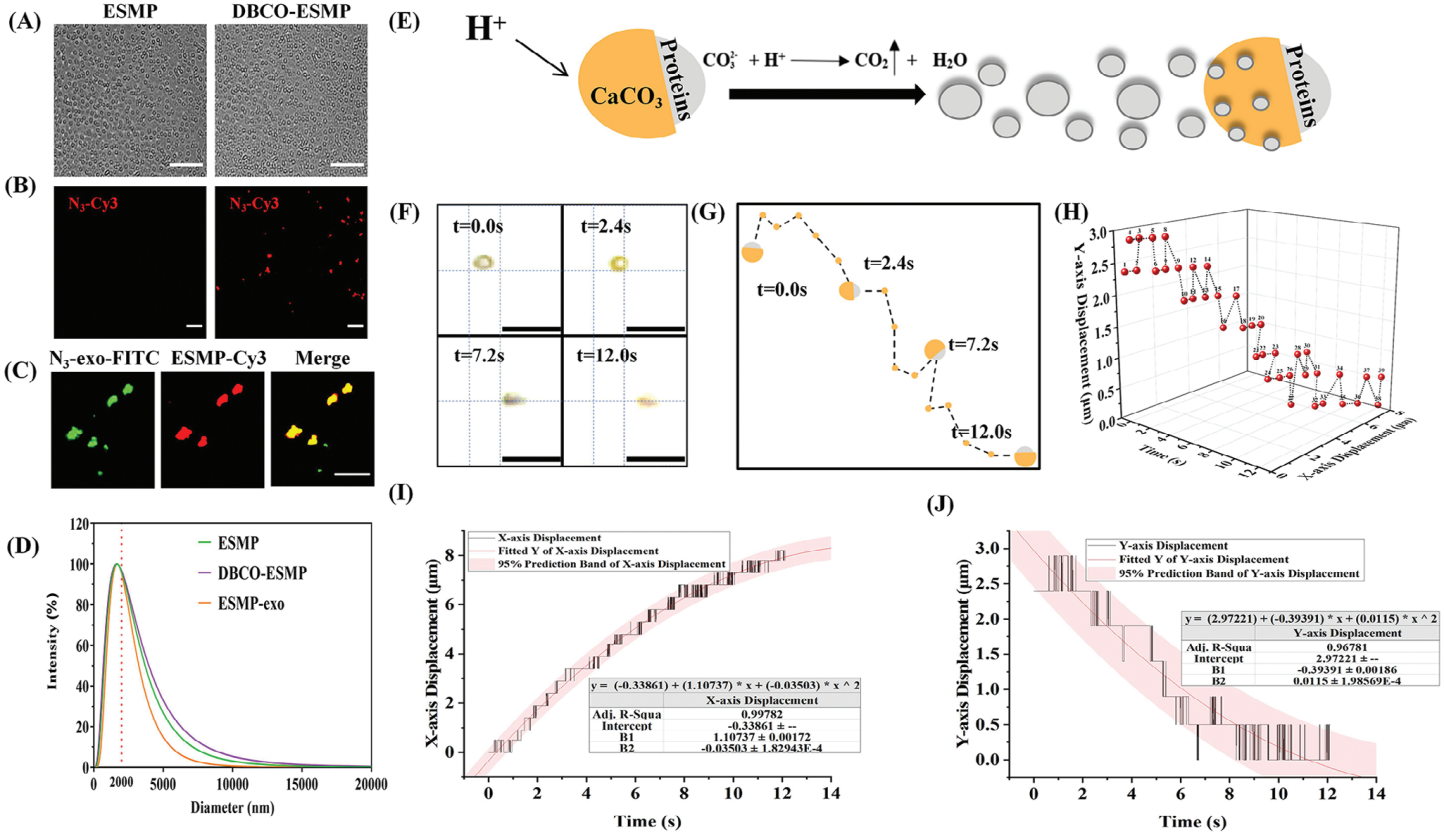
The CaCO_3_/proteins asymmetric microparticle driven by the acid‐calcium carbonate reaction. A,B) ESMP characterization. N_3_‐Cy3 probes (red) specifically conjugated to DBCO‐ESMP particles via a bioorthogonal reaction. Scale bar: 25 µm. C) Fluorescence images of DBCO‐ESMPs loaded with N_3_‐exosomes. N_3_‐exosomes pre‐labeled with DBCO‐FITC (N_3_‐exo‐FITC), and DBCO‐ESMPs labeled with N_3_‐Cy3 (ESMP‐Cy3). Scale bar: 20 µm. D) Particle size analysis. ESMP‐exo: a complex formed by DBCO‐ESMPs and N_3_‐exosomes through bioorthogonal reactions. E) Schematic diagram of the driving mechanism for the asymmetric particle. F) Time‐lapse images (from Video S6, Supporting Information) showing particle propulsion at 0, 2.4, 7.2, and 12 s. Scale bar: 10 µm. G) Motion trajectory of a CaCO_3_/proteins particle suspended in ultrapure water and driven by citric acid solution at pH 2. H) Particle trajectory changes over time. I,J) Motion analysis of the CaCO_3_/proteins particle in the X‐ and Y‐axis directions, respectively.

Microparticles of eggshell with the fibrous plasma membrane were prepared for DBCO‐NHS modification on the membrane side. As in **Figure** **5**A, under light microscopy, both the unmodified and DBCO‐NHS modified particles exhibit approximate circular shapes with uniform size. The fluorescence images show that DBCO‐NHS‐modified eggshell microparticles (DBCO‐ESMP) could be specifically labeled by N_3_‐Cy3 probes by a bioorthogonal reaction, indicating successful modification of eggshell microparticles (Figure 5B). We also found that N_3_‐Cy3‐labeled ESMP co‐localized with the DBCO‐FITC‐labeled exosomes (Figure 5C). And particle size analysis showed that the conjugates, ESMPs, and DBCO‐ESMPs have a diameter of ≈2 µm (Figure 5D).

### 2.6 Characterization of Eggshell Microparticle (ESMP) Motion

The self‐driving is asymmetrically propelled by carbon dioxide bubbles due to the calcium carbonate side of ESMPs reacting with acid (Figure 5E). The motion of ESMPs was captured using an ultra‐high‐speed camera. The ESMP suspension was injected into a microfluidic flow chamber where pH can be tuned by a micropump. Upon administration of a 1 M citric acid solution or a pH = 2 solution at a rate of 20 µl min^−1^, time‐lapse images revealed a considerable number of ESMPs undergoing rapid movement accompanied by size reduction (Videos S4 and S5, Supporting Information). In simulating the pH environment of MI, the solution with a pH of 5.0 was studied for the ESMPs movement, and we found the ESMPs moved at a relatively slower speed (Video S6, Supporting Information).

To rule out the effect of acid flow itself on the movement of ESMPs, a double reservoir glass slide with a connecting hydrophilic channel was custom‐made. The ESMP suspension was added to one reservoir, and the acid solution was then added to the other reservoir, flowing into the ESMP suspension along the connecting channel. The time‐lapse image shows that ESMP moved rapidly driven by a number of bubbles, when 1 m citric acid solution was added (Video S7, Supporting Information). To ensure that the experimental design exclude the influence of acid solution flow, we replace acid solution with double distilled water (ddH_2_O) as the driving solution. The particles ≈1 µm in diameter have no indication of movement (Video S8, Supporting Information). However, upon introducing a citric acid solution to pH of 2.0, the particle exhibited a remarkable movement (Video S9, Supporting Information). The findings confirm that ESMPs react with acid solution to produce gases, thereby to propel themselves forward.

The preliminary analysis of Video S9 (Supporting Information) shows that the position of the ESMP remained steady on both the X‐axis and Y‐axis after the 6663rd frame (Figure S5A,B, Supporting Information). As a result, the first 8000 frames were selected for further examination (Figure S5C,D, Supporting Information). The frame number was converted to time, and pixels were translated into their corresponding real‐world lengths. **Figure** **5**F depicts the positional changes of the ESMP at 0.0, 2.4, 7.2, and 12.0 s. Figure 5G,H presents the tracking path of the ESMP micromotor in water, indicating movement along both the X‐axis and Y‐axis. Further exploring the trajectory of ESMP micromotors, we find that their displacement along the X‐axis exhibits a significant quadratic function characteristic, conforming to the formula Y_1_ = −0.03503X^2 + 1.10737X–0.33861; whereas the displacement along the Y‐axis follows another quadratic function, given by the formula Y_2_ = 0.0115X^2–0.39391X + 2.97221 (Figure 5I–J). By taking the derivative of the displacement formulas, we successfully derive the velocity expressions. Specifically, the velocity formula along the X‐axis is Y'_1_ = −0.07006X + 1.10737, and the velocity formula along the Y‐axis is Y'_2_ = 0.023X – 0.39391. So, once triggered, the ESMP micromotor would gain a velocity of ≈1.2µm s^−1^ along the X‐axis and ≈0.4 µm s^−1^ in the Y‐axis direction. This discovery provides a strong mathematical basis for our deeper understanding of the movement characteristics of micro‐motor particles.

### 2.7 Characterization of Microneedle (MN) Patches

MN patches were manufactured on polydimethylsiloxane (PDMS) mold. The needle of PLGA ensures 1) sufficient hardness to allow penetrating into cardiac tissue and 2) a fast acid‐induced degradation. The effervescent base could be quickly released from the patch, leaving the needles in the heart. For comparison, we design the following 3 types of needles: 1) PLGA needle/base, 2) PLGA needles on an effervescent base (PLGA needle‐effervescent base), and 3) PLGA needles doped with ESMPs on an effervescent base (PLGA/ESMP needle‐effervescent base).

**Figure 6:**
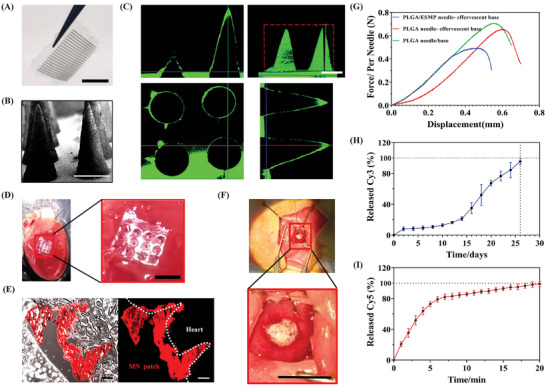
MN Patch Characterization and Degradation. A) A comprehensive view of the 20 × 20 MN patch. Scale bar, 1 cm. B) SEM image of the MN patch. Scale bar, 500 µm. C) 3D and three‐view images of an MN patch loaded with FITC. Scale bar: 500 µm. D) MN patch inserted into a rat heart ex vivo. The inset (red solid box) showing a higher magnification of the inserted area. Scale bar, 2 mm. E) Fluorescent images displaying the complete insertion of Cy3‐labeled MN needle tips into the rat heart. Scale bar, 200 µm. F) In vivo degradation of the effervescent base. The inset (red solid box) is shown at a higher magnification in the right panel. Scale bar, 2.5 cm. G) Mechanical strength of the single needle. H,I) In vitro degradation of the needles and the effervescent base. Data points represent the mean ± SD (*n* = 3). Error bars indicate SD.

All patches are in 20 × 20 array with a needle height of 1000 µm (**Figure** **6**A,B). For visibility, FITC‐labelled patches were designed to present that the needles are in smooth and conical forms (Figure 6C). The MN patch can be conformably inserted into rat heart tissue (Figure 6D) as from fluorescence image of the frozen sectioned cardiac tissue (Figure 6E). After adding a small amount of saline, the effervescent base produced amounts of bubbles and rapidly degraded in minutes (Figure 6F; Video S10, Supporting Information). The single‐needle strength of the PLGA needle/base group reaches the maximum of ≈0.7 N, while the PLGA/ESMP needle effervescent base has the weakest of ≈0.5 N (Figure 6G). This is because PLGA works as both needle and base without interfacial joint concern. In vitro degradation testing of Cy3‐labeled MN patches reveals a degradation of ≈24% after 14 days, followed by a rapid degradation phase where ≈4% remained after 26 days. (Figure 6H). The Cy5‐labeled effervescent base experienced 75% degradation within 5 min and disappeared after 20 min (Figure 6I).

### 2.8 Assessing Therapeutic Efficacy

**Figure 7:**
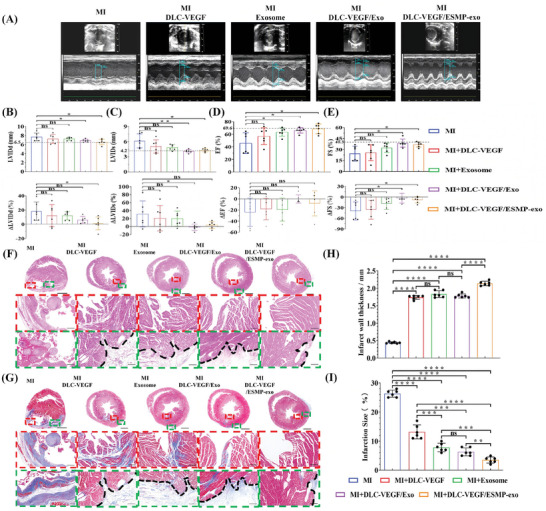
Assessing rat cardiac function and morphology 28 days After MI. A–E) Assessing rat cardiac function via ultrasound detection. Relative changes analysis was conducted based on the LVIDd, LVIDs, EF, and FS values of normal rats (average values of 5 rats). F,G) Examining rat cardiac morphology through H&E and Masson staining. Scale bar, 2000 µm. Insets outlined by red and green dashed boxes were displayed at higher magnification in the bottom row. Scale bar, 250 µm. The black dashed line indicated the MN patch insertion position. H,I) Quantification of morphological parameters, including infarct wall thickness and infarct area, from Masson‐stained images using ImageJ software. All data were presented as mean ± SD (*n* = 6). Comparisons between two groups were performed using two‐tailed unpaired Student's t test, and statistical significance was indicated by the asterisks (*) above the lines. NS indicates *p* > 0.1234. *p < 0.0332, and **p < 0.0021. ***p < 0.0002, and ****p < 0.0001.

Acute myocardial infarction was induced in rats by ligating the left anterior descending coronary artery (Figure S6A, Supporting Information). Thirty minutes following the MI, ultrasound and electrocardiographic (ECG) examinations were conducted. The ultrasound images reveal a considerable decline in the left ventricular beat rate in the ligated rats compared to before the ligation. Furthermore, ECG images display ST‐segment elevation (Figure S6C,D, Supporting Information). Subsequently, the rat heart was extracted for triphenyl tetrazolium chloride (TTC) staining, which exhibited gray in the infarcted part (Figure S6B, Supporting Information). The MI‐affected rats were divided into five groups: the control group (MI), and four treatment groups consisting of MN patches loaded with DLC‐VEGF nanoparticles (DLC‐VEGF), MN patches containing exosomes (Exosome), MN patches combining DLC‐VEGF nanoparticles and exosomes (DLC‐VEGF/Exo), and MN patches incorporating DLC‐VEGF nanoparticles and ESMP‐exo complex (DLC‐VEGF/ESMP‐exo). Ultrasound assessments were performed after 28 days to assess the therapeutic efficacy. We found that the left ventricular anterior wall in the MI group exhibits virtually no pulsation. Among all the treatment groups, the DLC‐VEGF/ESMP‐exo group demonstrates the most significant recovery effect (**Figure** **7**A). The MI rats exhibited a notably expanded left ventricular end‐diastolic diameter (LVDd) and end‐systolic diameter (LVDs), with measurements of 7.708 ± 0.8563 mm and 6.166 ± 1.392 mm, respectively. These dimensions were substantially greater than the normal group's 6.500 and 4.180 mm (Figure 7B,C). In the DLC‐VEGF and Exosome groups, the LVDd and LVDs were elevated beyond normal levels, registering at 7.278 ± 1.011 mm, and 7.28 ± 0.3486 mm, and 5.059 ± 1.333 mm and 4.864 ± 0.5658 mm, respectively. Notably, the DLC‐VEGF/Exo and DLC‐VEGF/ESMP‐exo groups demonstrated nearly indistinguishable LVDd and LVDs values akin to those of normal rats, with readings of 6.906 ± 0.318 mm and 6.592 ± 0.596 mm, and 4.112 ± 0.338 mm and 4.334 ± 0.349 mm, respectively. All groups exhibited left ventricular ejection fraction (EF) and fractional shortening (FS) values significantly below the normal range, with averages at 69.64% and 40.25%, respectively. The MI group demonstrated the most profound deficits, with EF and FS readings of 45.91% ± 16.51% and 24.52% ± 10.24%. Notably, the exosome group exhibited superior EF and FS values compared to the DLC‐VEGF group, with respective averages of 57.03% ± 13.30% versus 63.56% ± 6.54% for EF, and 25.80% ± 10.89% versus 32.50% ± 6.46% for FS. Furthermore, the EF and FS values in the DLC‐VEGF/Exo and DLC‐VEGF/ESMP‐exo groups showed a marked improvement, reaching 66.15% ± 4.250% and 68.72% ± 7.823% for EF, and 38.82% ± 5.527% and 36.97% ± 3.585% for FS, respectively.

### 2.9 Pathological Staining of Cardiac Tissue

Four weeks after MI, The HE staining shows a considerable thinning of the rat ventricular wall in the MI group, resulting in ventricular dilation and deformation (Figure 7F). The cardiomyocyte layers form an almost regular ellipsoid, with a diameter ranging from ≈150 to 500 µm, separating from the ventricular wall. The overall ventricular structure remains stable in the DLC‐VEGF and exosome groups, with no significant changes observed. However, in the border and inner regions, the cardiomyocyte nuclei appear disorganized and partially cluster, suggesting potential myofiber degradation and cardiomyocyte detachment. In the DLC‐VEGF/Exo group, the nuclei are arranged neatly in the border region, and the myofibers present a regular pattern of distribution. Nonetheless, the myofibers in the inner region appear disorganized and tend to break down. In contrast, the cardiomyocytes in the DLC‐VEGF/ESMP‐exo group exhibits a regular nuclear arrangement and consistent gaps in both the border and inner regions of the MI. Furthermore, the myofibers maintained a consistent orientation, showing no signs of decomposition. Figure 7G illustrates the morphology and fibrosis of rat heart tissues post‐Masson staining. The measurement revealed that the ventricular wall in the MI group exhibited the most remarkable thinning, with a measurement of just 0.443 ± 0.025 mm (Figure 7H). In contrast, the treatment groups demonstrated a significant enhancement in ventricular wall thickness. Among these, the DLC‐VEGF, Exosome, and DLC‐VEGF/Exo groups exhibited similar thicknesses of 1.742 ± 0.055 mm, 1.834 ± 0.097 mm, and 1.782 ± 0.06 mm, respectively. However, the DLC‐VEGF/ESMP‐exo group stood out with a notably thicker ventricular wall, measuring 2.141 ± 0.064 mm. Additionally, the DLC‐VEGF/ESMP‐exo group showed the most significant improvement in infarct size, which was only 3.500% ± 1.049% (Figure 7I). Although the degree of fibrosis in the other three treatment groups was alleviated compared with the MI group (26.33% ± 1.211%), the presence of fibrosis still cannot be ignored. Among these, the fibrosis phenomenon was more pronounced in the DLC‐VEGF group, with specific values of 13.170% ± 2.401%. There was no significant difference in the degree of fibrosis between the Exosome group and the DLC‐VEGF/Exo group, with values of 7.833% ± 1.472% and 6.333% ± 1.366%, respectively.

### 2.10 Exploring the Therapeutic Mechanism of Self‐Driven Combined Drug Delivery MN Patches

**Figure 8:**
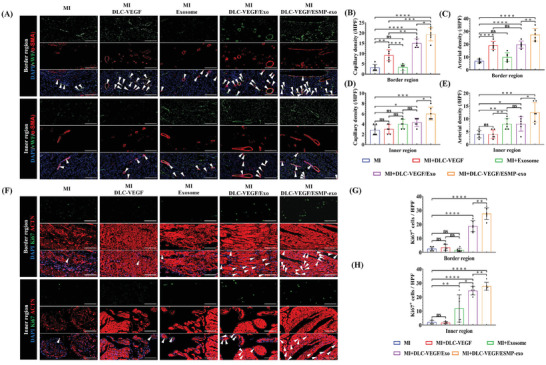
Exploring vascular generation and myocardial cell regeneration through fluorescence staining. A) Representative immunofluorescent images of vWF (green) and α‐SMA (red) in the border and inner regions of the infarcted area. Arrows indicate the areas positive immunofluorescence staining. Scale bar: 200 µm. B–E) Quantification of capillary density and arterial density in the inner and border regions using ImageJ analysis (*n* = 6). F) Detection of Ki67+ expression (green) in the border and inner regions of the infarcted area. Arrows indicate the areas positive immunofluorescence staining. Scale bar: 100 µm. G,H) Ki67+ cells per high power field (HPF) evaluated by ImageJ software (*n* = 6). All data were presented as mean ± SD. Comparisons between two groups were performed using two‐tailed unpaired Student's t test, and statistical significance was indicated by the asterisks (*) above the lines. NS indicates *p* > 0.1234. *p < 0.0332, and **p < 0.0021. ***p < 0.0002, and ****p < 0.0001.

We employed antibodies against vascular hemophilia factor (vWF) and α‐smooth muscle actin (α‐SMA) to distinguish endothelial cells and vascular smooth muscle cells, respectively. Tubular structures stained with fluorescent antibodies were identified as vessels. The capillary density was calculated by counting the number of vWF‐positive vessels per high‐power field of view (HPF), while the arterial density was assessed by counting the number of α‐SMA‐positive vessels per HPF (**Figure** **8**A). As shown in Figure 8B, the capillary density in the border region of the MI group was comparable to that of the Exosome group, with values of 3.167 ± 1.602 and 3.333 ± 1.506, respectively, both of which were significantly lower than those in the other groups. The DLC‐VEGF/ESMP‐exo group had the highest capillary formation, reaching 19.330 ± 3.204, which was significantly better than those of the DLC‐VEGF/Exo group and the DLC‐VEGF group at 15.000 ± 2.098 and 9.167 ± 2.639, respectively. Similarly, the DLC‐VEGF/ESMP‐exo group also performed best in arterial angiogenesis, up to 27.330 ± 5.007. The arterial density in the DLC‐VEGF/Exo group was slightly higher than that in the DLC‐VEGF group, with values of 19.670 ± 2.733 and 19.000 ± 3.162, respectively. At the same time, the Exosome group also had a slightly higher arterial density than the MI group, with specific values of 9.833 ± 3.430 and 6.833 ± 1.472 (Figure 8C). In the inner region, the capillary density of the MI group was similar to that of the DLC‐VEGF group, with specific values of 2.833 ± 0.983 and 3.000 ± 0.894, respectively, which were significantly lower than those in the other groups (Figure 8D). The capillary density in the Exosome group was roughly equivalent to that in the DLC‐VEGF/Exo group, with values of 4.000 ± 0.894 and 4.333 ± 0.817, respectively. However, those values were still significantly lower than that in the DLC‐VEGF/ESMP‐exo group, which had a capillary density as high as 6.000 ± 1.265. Additionally, the DLC‐VEGF/ESMP‐exo group exhibited an exceptionally rich arterial vascularization, with a density of up to 12.500 ± 3.834 (Figure 8E). Although there was no significant difference in arterial density between the Exosome group and the DLC‐VEGF/Exo group (8.000 ± 2.098 and 8.000 ± 2.828), it was almost twice that of the DLC‐VEGF group and the MI group (4.000 ± 1.673 and 4.000 ± 1.414).

Subsequently, we conducted ACTN2 staining and Ki67 expression analysis on rat heart tissue sections (Figure 8F). Ki67 serves as a marker for cell proliferation.^[^ ^24^ ^]^ The proliferative capacity of myocardial cells was determined by counting the number of Ki67‐positive cells per HPF. Figure 8G illustrates a comparative analysis of Ki67‐positive cell numbers across different groups. In border regions, the MI group exhibited a slightly higher compared with the Exosome group (2.500 ± 1.378 vs 1.667 ± 1.211); however, it was notably lower than that of the DLC‐VEGF group (3.500 ± 2.074). A remarkable enhancement in Ki67‐positive cell numbers was observed in the DLC‐VEGF/Exo and DLC‐VEGF/ESMP‐exo groups, with values of 18.670 ± 3.882 and 27.670 ± 4.179, respectively. The latter group demonstrated a more pronounced increase when contrasted with the former. In inner regions, the Ki67‐positive cell numbers in the MI group and the DLC‐VEGF group were nearly identical (2.000 ± 1.265 and 1.667 ± 0.817), both significantly lower than that in the Exosome group (11.83 ± 9.704) (Figure 8H). Despite the higher count in the DLC‐VEGF/Exo group (24.500 ± 3.017) compared with the Exosome group, it remained inferior to the number in the DLC‐VEGF/ESMP‐exo group (27.670 ± 2.805).

Furthermore, MI triggers an inflammatory response, characterized by a significant infiltration of CD68‐positive macrophages into the damaged cardiac tissue.^[^ ^25^ ^]^ In the border and inner regions of infarction, pro‐inflammatory M1‐type macrophages intertwined with anti‐inflammatory M2‐type macrophages. We identified macrophage infiltration by performing immunofluorescence staining on frozen sections of rat heart tissue 28 days post MI. For the identification of M1‐type macrophages, we employed fluorescence colocalization of antibodies against CD68 with inducible nitric oxide synthase (iNOS) or tumor necrosis factor‐α (TNF‐α); while for the recognition of M2‐type macrophages, we utilized fluorescence colocalization of antibodies against CD68 with arginase 1 (ARG1) or transforming growth factor‐β (TGF‐β). The fluorescence analysis revealed a substantial accumulation of M1‐type and M2‐type macrophages in the border and inner regions of the infarcted area in the untreated and the single treatment groups, including the MI, DLC‐VEGF, and exosome groups (Figure S7A,B, Supporting Information). However, while the macrophage infiltration persisted prominently in the border region of the DLC‐VEGF/Exo dual‐drug‐loaded group, it was notably reduced within the inner region. In the self‐driven dual‐drug‐loaded group, the DLC‐VEGF/ESMP‐exo group, the infiltration was significantly reduced in the border region, with nearly undetectable infiltration in the inner region. Furthermore, the macrophage infiltration within the infarcted rat heart was not obviously different from that observed in the non‐infarcted rat.

### 2.11 ELISA Detection of Rat Serum

Serial serum samples were collected every 7 days post‐MI for subsequent comprehensive analysis, including alanine aminotransferase (ALT), aspartate aminotransferase (AST), blood urea nitrogen (BUN), and creatinine. The findings revealed that the four indicators in the experimental groups exceeded those in the normal rats. In the ALT assessment, the DLC‐VEGF group demonstrated the highest reading (Figure S8A, Supporting Information). Despite slightly lower values than the MI group, the DLC‐VEGF/ESMP‐exo group still exceeded the Exosome and DLC‐VEGF/Exo groups. For the AST detection, the readings for the DLC‐VEGF/ESMP‐exo group were virtually identical to those of the Exosome group. These values were higher than the MI group's, but lower than those of the DLC‐VEGF and DLC‐VEGF/Exo groups (Figure S8B, Supporting Information). In the BUN assessment, the results exhibited a negligible discrepancy between the MI and DLC‐VEGF groups. The readings in the DLC‐VEGF/ESMP‐exo, Exosome, and DLC‐VEGF/Exo groups were virtually identical, exhibiting a notable decline compared to the MI and DLC‐VEGF groups (Figure S8C, Supporting Information). For the creatinine detection, the DLC‐VEGF/ESMP‐exo group demonstrated slightly lower readings than the DLC‐VEGF group, yet significantly higher than the MI group. The MI group's values were more aligned with those of the Exosome and DLC‐VEGF/Exo groups (Figure S8D, Supporting Information). To further investigate the potential impact of ESMPs on biosafety, we established two additional experimental groups, including normal rats with DLC‐VEGF/ESMP‐exo MN patches (normal + DLC‐VEGF/ESMP‐exo) and MI rats with ESMP‐MN patches (MI + ESMP). The results demonstrated that the ALT, AST, and creatinine levels in both new groups were higher than those in the MI group, with a similar changing trend (Figure S9A–C, Supporting Information). In the BUN test, the MI group displayed the highest values (Figure S9D, Supporting Information).

Given VEGF's substantial role in angiogenesis,^[^ ^18^ ^]^ monitoring fluctuations in blood VEGF levels is essential. The study uncovered that serum VEGF concentration in rats following MI were elevated compared to normal rats, irrespective of treatment. Among the experimental groups, the DLC‐VEGF/ESMP‐exo group demonstrated the most prominent VEGF expression. During the initial three weeks, the VEGF levels in this group remained parallel to those in the DLC‐VEGF group. Nonetheless, in the fourth week, the VEGF expression in the DLC‐VEGF group surpassed that of the DLC‐VEGF/ESMP‐exo group. A similar trend was observed in the Exosome group, with levels slightly higher than the DLC‐VEGF/Exo group in the first three weeks but lower in the fourth week. Although VEGF levels in the MI group slightly increased in the first two weeks, they plummeted to the lowest among the experimental groups in the subsequent two weeks (Figure S10A, Supporting Information). Furthermore, the VEGF levels in the Normal + DLC‐VEGF/ESMP‐exo group were lower than those in the MI group during the first two weeks, but they began to rise and exceeded the MI group in the third week (Figure S10B, Supporting Information). In contrast, VEGF levels in the MI+ESMP group remained the highest throughout the four‐week period.

## 3 Discussion

The abrupt decrease in pH within infarct zones triggers acidosis in cardiomyocytes. The severe acidosis can lead to cell necrosis, primarily due to the activation of lysosomes and lipases, disruption of ionic gradients, and inhibition of enzyme reactions.^[^ ^5^ ^]^ And the cessation of blood flow in infarct regions erects insurmountable barriers to the delivery and diffusion of therapeutic agents. To enhance the efficacy of exosomes in MI treatment, researchers have proposed many strategies, including gene editing, peptide chain modification, drug pretreatment, and hypoxia stimulation to enhance their homing and cardioprotective capabilities.^[^ ^26^ ^]^ Well‐designed exosomes are expected to effectively rescue myocardial cells in the infarcted regions; however, the outcomes have not met expectations. This suggests a crucial oversight: the weakly acidic microenvironment poses a direct threat to myocardial cells. Therefore, raising the local pH should be the first step in MI treatments.

In our research, the administration of exosomes or eggshell particles alone proved insufficient to rescue cardiomyocytes under hypoxic‐acidic conditions (Figure 2A–C). Within the Exo group, the lack of pH regulation resulted in severe shrinkage of myocardial cells; and in the ESMP group, despite a notable rise in pH, myocardial cells exhibited extensive aggregation. However, the ESMP + Exo group demonstrated a remarkable dual benefit: not only was the pH further elevated, but the myocardial cells also maintained intact cellular morphology. Considering the pivotal role of intracellular calcium levels in modulating cardiomyocyte contractility,^[^ ^3^ ^]^ and the detrimental consequences of calcium overload, which can precipitate cardiomyocyte apoptosis,^[^ ^27^ ^]^ we conducted an in‐depth study of the intracellular calcium ion levels (Figure 2D,E). Our research revealed that, under the regulation of exosomes, the calcium ion levels in the ESMP + Exo group exceeded those in the ESMP group, despite both groups receiving supplementary calcium; however, it is noteworthy that the ESMP + Exo group remained below the levels observed in the Normal group exposed to 20% oxygen conditions, thus to ensure the prolonged survival of myocardial cells. Consequently, we concluded that ESMPs effectively regulate the pH of the acidic medium, and, in conjunction with exosomes, significantly enhance the resilience of cardiomyocytes in hypoxic‐acidic environments.

Human heart organoids were subjected to hypoxic‐acidic conditions with the pH of ≈5.8, replicating the microenvironment following MI.^[^ ^16a^ ^]^ The HCO MI model breaks through species limitations,^[^ ^28^ ^]^ endowing the research outcomes with clinical reference value. Our findings revealed that there was no statistically significant distinction in the reduction of beat frequency between the ESMP + Exo group and the Normal group, and that myocardial cells were effectively rescued, concurrently preserving their robust proliferative potential (Figure 2I–K). Given the negligible decline in pH within the Normal group and the ESMP + Exo group's pH acceptably below the baseline (Figure 2F), the observed decrease in beating frequency and contraction amplitude in both the ESMP + Exo and Normal groups (Figure 2G,H; Videos S1 and S3, Supporting Information) should be attributed to the heart's intrinsic self‐regulation mechanism initiated by the hypoxic environment. Particularly, HCOs in the AM group exhibited a significant decline in beating frequency, with a decrease of up to 84% ± 21.93%, and even an abnormal phenomenon of beating cessation (Video S2, Supporting Information). These outcomes affirmed that in the MI treatment, the paramount objective is to improve the weakly acidic microenvironment, thereby significantly enhancing the therapeutic efficacy of exosomes.

To further validate the in vivo therapeutic effect, the initial hurdle lies in the efficient delivery of exosomes. Our in vitro studies have demonstrated that, upon activation by an acidic solution, ESMPs obtained an initial velocity of ≈1.26 µm s^−1^ and exhibited a composite motion along the X‐axis and Y‐axis, which conforms to quadratic equations (Figure 5E–J). Moreover, chemically modified protein membranes could be laden with metabolically modified N_3_‐exosomes through bioorthogonal reactions (Figure 5A–D). Capitalizing on these insights, we developed a self‐driven dual‐drug‐loaded MN patch, targeting MI‐acidic microenvironment, to delivery exosomes. Recognizing the pivotal role of VEGF in early vascular reconstruction post‐MI,^[^ ^18^ ^]^ we encapsulated VEGF lipid particles in the effervescent base of MN patches.

The self‐driven dual‐drug‐loaded MN patch is composed of two parts: the effervescent base and the PLGA microneedle, each responsible for the release of VEGF lipid nanoparticles and exosome‐loaded self‐driven particles, respectively. VEGF liposomes are rapidly released from the effervescent base within just 20 min (Figure 6I), quickly taken up by endothelial cells (Figure 4F), and effectively stimulate the migratory activity of endothelial cells (Figure 4G–K), greatly promoting the early reconstruction process of vascular network. In contrast to VEGF, exosomes exhibit a more comprehensive yet gradual effect on MI repair, facilitating not only vascular reconstruction^[^ ^7^ ^]^ but also the proliferation of cardiomyocytes^[^ ^6^ ^]^ and the modulation of inflammation.^[^ ^8^ ^]^ This sustained release through the microneedle over an approximate duration of 26 days (Figure 6H) ensures a long‐lasting reparative effect (Figure 3I–O). The synergistic interplay of VEGF and exosomes facilitates a 3D reconstruction of the vascular network from the outside in. And on the basis of the self‐propelled particles' pH restoration, exosomes effectively rescue distal cardiomyocytes, thereby preventing the occurrence of fibrosis in the later stage of myocardial infarction.

In the MI rats, a dramatic reduction in ventricular wall thickness was observed, merely to 0.4432 mm, accompanied by the detachment of substantial myocardial tissue clumps, ranging in diameter from 150 to 500 µm, from the ventricular wall (Figure 7F–I). In the single treatment groups, while no such severe ventricular remodeling was evident, myocardial tissue exhibited a tendency to detach from the ventricular wall to varying degrees, and fibrosis was noted both in the border and the inner zones. In contrast, the DLC‐VEGF/ESMP‐exo group showed a tightly and orderly arranged array of cardiomyocytes, with no fibrosis detected in the inner zone. The slight fibrosis observed on the myocardial surface was attributed to needle retention. Immunofluorescence analysis revealed that the DLC‐VEGF/ESMP‐exo group exhibited abundant microangiogenesis both within and outside the infarct regions (Figure 8A–E), and the regenerative cardiomyocytes were successfully preserved. The detection of inflammation in the infarcted area further revealed that the self‐driven dual‐drug‐loaded MN patch effectively inhibited the infiltration of M1‐type and M2‐type macrophages within the border and inner regions, thereby preventing the fibrotic process and providing a strong guarantee for the recovery of cardiac function post MI (Figure S7, Supporting Information). Furthermore, liver and kidney metabolic markers detection indicated that the MN patch possessed excellent biocompatibility (Figures S8 and S9, Supporting Information).

**Figure 9:**
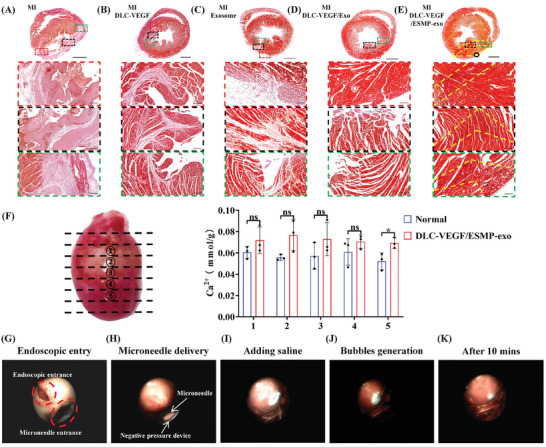
In vivo calcium ion tracing and expanded applications of the two‐stage drug‐loaded patch in pig models. A–E) Alizarin red staining of rat cardiac tissue slices from the untreated and treated groups. Scale bar: 2000 µm. The insets outlined by red, black, and green dotted boxes are shown at higher magnification in the bottom row. Scale bar: 200 µm. F) Determination of calcium ion content in rat heart tissues. All data were presented as means ± SD (*n* = 3). Comparisons between two groups were performed using two‐tailed unpaired Student's t test, and statistical significance was indicated by the asterisks (*) above the lines. NS indicates *p* > 0.1234, and *p < 0.0332. G–K) Minimally invasive delivery of an MN patch to the pig heart with the aid of endoscopic technology. Images from Video S12 (Supporting Information).

Our comprehensive investigation delved into the distribution of calcium ions within the myocardium of rat hearts. The findings revealed a distinctive fan‐shaped pattern of calcium ion distribution in hearts treated with the self‐driven, dual‐drug‐delivering MN patch, emanating from the MN injection site and radiating extensively into deeper layers of myocardium (**Figure** **9**A–E). The calcium ion distribution was not observed in other treatment groups or in healthy rats. Moreover, through the detection of calcium ion concentration in infarct regions, we ascertained that the calcium ion concentration across all segmented regions in the DLC‐VEGF/ESMP‐exo group was significantly higher than that in the normal rats (Figure 9F). These data conclusively confirmed ESMPs, propelled by the weak acidic microenvironment, were capable of delivering exosomes to each affected region, ensuring comprehensive and profound efficacy. Furthermore, we have validated the feasibility of deploying the self‐driven, dual‐drug‐delivering MN patch in rabbit and pig hearts via minimally invasive thoracoscopic surgery (Figure 9G–K; Figure S11, Videos S11 and S12, Supporting Information), signifying substantial potential for future clinical application.

In this study, the HCOs employed did not form a comprehensive vascular network, however, it is well‐known that the reconstruction of vascular network plays a crucial role in the myocardial repair. In light of this, the MI model of HCOs aimed to verify the protective and reparative effects of combined therapy on myocardial cells. To augment the retention of exosomes within the infarct zone, we constructed the PLGA needles designed to persist in myocardial tissue for an extended duration of ≈26 days. The design, like a two‐edged sword, not only achieves the sustained release of exosomes but also leaves irreversible fibrosis marks on the myocardial surface. Furthermore, despite our successful modification of exosomes with azides via cellular metabolism, their functions were not further refined, which may compromise the full realization of their therapeutic efficacy in MI. The Thoracoscope‐assisted MN patch implantation technique, as an innovative medical approach with great potential for clinical application, holds a promising future. However, in practice, we must comprehensively consider many factors to ensure the safety and efficacy of the surgery. For instance, the lower anesthetic tolerance among MI patients necessitates a stringent risk assessment for general anesthesia. Moreover, the reduced surgical incision in clinical procedures, in contrast to experimental models, underscores the necessity for expert thoracoscopic skills. Despite the MN patch's ongoing need for further clinical trials, this study has provided a good reference example. We are optimistic that, with the relentless efforts of medical translational researchers and clinicians, MN patches will be widely used in minimally invasive clinical surgery, bringing blessings to patients.

## 4 Experimental Section

### Animals

All animals were housed in a strictly controlled environment to ensure the health and well‐being of the animals. In the experiment, the rats were anesthetized by intraperitoneal injection of 30mg kg^−1^ of sodium pentobarbital solution and ventilated with the help of endotracheal intubation. The Panamanian pigs received intramuscular injection of 30mg kg^−1^ sodium pentobarbital solution to induce anesthesia, followed by ventilation via endotracheal intubation and the application of 2% isoflurane to sustain their anesthetic state. The New Zealand white rabbits underwent a comparable process, injected with 30 mg kg^−1^ sodium pentobarbital, ventilated identically, and maintained under anesthesia with 1% isoflurane. All protocols follow the Animal Research Ethics Guidelines and have been approved by the Animal Center of the Army Medical University, China, under the approval number AMUWEC20223087.

### Statistics

The data were analyzed using GraphPad Prism 8.0 (San Diego, CA, USA) and presented as the means ± SD. For comparisons between samples, a single‐sample t test was employed. To assess differences between two groups, a two‐tailed Student's t test was used. Moreover, one‐way analysis of variance (ANOVA) was performed for comparisons involving more than two groups. Statistical significance was set at *p < 0.0332, and **p < 0.0021. ***p < 0.0002, and ****p < 0.0001.

## Conflict of Interest

The authors declare no conflict of interest.

## Author Contributions

F.F.W., Z.L.X., and F.Y.Z. contributed equally to this work. M.X., C.H.Z., and F.F.W. conceived the hypotheses, methods, and applications; F.F.W., Z.L.X., F.Y.Z., D.C.Y., M.K., X.Z., X.H.Z., J.T., Y.L., Y.S.H., K.X. performed the experiments; F.F.W., Z.L.X., F.Y.Z. and K.X. analyzed the data; F.F.W., Z.L.X., M.X., and C.H.Z. wrote the manuscript; and all authors discussed results and commented on the manuscript.

## Supporting information



Supporting Information

Supplemental Video 1

Supplemental Video 2

Supplemental Video 3

Supplemental Video 4

Supplemental Video 5

Supplemental Video 6

Supplemental Video 7

Supplemental Video 8

Supplemental Video 9

Supplemental Video 10

Supplemental Video 11

Supplemental Video 12

## Data Availability

The data that support the findings of this study are available from the corresponding author upon reasonable request.
