# Supplementary material for: Cardiac Organoid Model Inspired Micro‐Robot Smart Patch to Treat Myocardial Infarction
Source: Adv Mater. 2025 Mar 6;37(26):2417327. doi: 10.1002/adma.202417327 (PMC12232214; doi:10.1002/adma.202417327)
Supplement: Supplementary file 1 — Supporting Information [file ADMA-37-2417327-s012.docx]

Supplementary Information

**Cardiac Organoid Model Inspired Micro-robot Smart Patch to Treat Myocardial Infarction**

**1. Materials and Methods**

- 1. **Establishing a hypoxic-acidic model of cardiomyocytes.** According to the standard components of the DMEM/F12 culture medium, we dissolved 17.49 mM of D-glucose (Beyotime, China) in physiological saline and adjust the solution's pH to about 5.8 to prepare a weakly acidic medium (AM). When the H9C2 cells reached to approximately 90% confluence in a six-well plate, we removed the supernatant and washed the cells twice with PBS. Afterwards, 2 mL of DMEM/F12 or AM was added to each well. Cells were subjected to aerobic and anaerobic treatments. The aerobic treatment was carried out under standard cell culture conditions, maintaining an atmosphere of approximately 20% oxygen. The anaerobic treatment was conducted within a custom-engineered anaerobic chamber, which was purposely filled with a gas mixture of 95% nitrogen and 5% carbon dioxide to create an oxygen-deprived environment. The aerobic treatment was divided into a control group (20% O_2_-DMEM/F12) and an acidic medium group (20% O_2_-AM). The anaerobic treatment included a control group (0% O_2_-DMEM/F12), an acid medium group (0% O_2_-AM), an eggshell microparticle treatment group (0% O_2_-AM+ESMP), an exosome treatment group (0% O_2_-AM+Exo), and an eggshell microparticle and exosome combined treatment group (0% O_2_-AM+ESMP+Exo). The concentration of eggshell microparticles was set at 185.51 mg/mL and the concentration of exosomes was 60 mg/mL. Cell supernatants were collected and measured using a pH meter (FiveEasy Plus, Switzerland). We used the Live/Dead Cell Staining Kit (Proteintech, China) to assess the cardiomyocyte viability, and performed fluorescence quantification analysis by ImageJ software. To analyze the accumulation of calcium ions in cardiomyocytes after anaerobic treatment, we supplemented AM with 1.0507 mM calcium chloride to match the concentration in DMEM/F12 medium. We utilized the Fluo-4 Calcium Ion Detection Kit (Beyotime, China) to measure the intracellular calcium concentration in cardiomyocytes, and performed fluorescence quantification analysis by ImageJ software. To ensure the comparability of the quantitative analysis of fluorescence signals, we strictly controlled the consistency of imaging conditions for the laser confocal microscope (Leica, Germany). The excitation wavelength was set to 488 nm, and the spectral range for detection was adjusted from 498 nm to 739 nm. The gain was set to 2.5, with a compensation value of 0.
  2. **Establishing a hypoxic-acidic model of HCOs.** Human induced pluripotent stem cells (hiPSCs) originated from the Cell Bank of the Chinese Academy of Sciences Typical Culture Collection Committee. The hiPSC suspension was seeded into a ultra-low adhesion U-shaped 96-well plate (Corning, USA) with 1×10^4^ cells per well. Subsequently, a 5-minute centrifugation at 100g was performed to allow the cells to settle and aggregate at the bottom of the culture plate. After cells aggregated into clumps, the induction culture was carried out according to the guidance of the Cardiac Organoid Culture Kit (M102-24, JKmed, China). When the suspended cell clusters exhibited regular beats, it indicated the successful construction of human cardiac organoids. Post-induction, the cardiac organoids were incubated in a maintenance medium. The medium was formulated by blending DMEM/F-12 (Gibco, USA) with Neurobasal (Gibco, USA) in a 1:1 ratio, supplemented with an additional 2 ng/mL of VEGF-A (Gibco, USA) and 20 mM of glutamine (Peprotech, USA). The acidic medium (AM) consisted of 2 ng/mL of VEGF-A and 20 mM glutamine in physiological saline, adjusted the pH to approximately 5.8.

HCOs exposed to AM were placed within a culture chamber supplemented with 5% carbon dioxide and 95% nitrogen to replicate the hypoxic-acidic environment post-MI. Initial experiments conducted in a standard cell incubator revealed that HCOs ceased beating within 20 minutes under acidic stimulation. Based on above findings, we subjected the HCOs to hypoxic-acidic conditions and administered corresponding treatments. In the experiment, cardiac organoids were randomly assigned to three different treatment groups: the control group (Normal) incubated with maintenance medium, the treatment group (AM) with acid medium, and the experimental group (AM+ESMP+Exo) receiving combined treatment with eggshell microparticles and exosomes. We recorded changes in the beating frequency of the organoids using an optical microscope (Leica, Germany). Furthermore, we evaluated the cell viability and proliferation capacity of cardiac organoids. Cardiac organoids were randomly assigned to three experimental groups: the control group (Normal) incubated with maintenance medium under standard culture conditions, the treatment group (AM) exposed to hypoxic-acidic conditions, and the experimental group (AM+ESMP+Exo) receiving combined treatment of eggshell particles and exosomes under hypoxic-acidic conditions. Following the corresponding treatments, the cardiac organoid specimens underwent immunofluorescence staining. Images were taken with a Leica confocal microscope and subsequent analysis performed with ImageJ software.

- 1. **Immunostaining of HCOs.** Following fixation with 4% paraformaldehyde (Beyotime, China) overnight, specimens were washed three times with a washing buffer (V11311, CytoVista, USA), each lasting at least 30 minutes. Next, the organoids were placed in antibody penetration buffer (V11309, CytoVista, USA) and incubated overnight on a 4°C shaker. After washed three times, the organoids were transferred to blocking solution (V11307, CytoVista, USA) and continued to be incubated overnight at 4°C. The primary antibodies, including rabbit anti-Ki67 (1:100, ab15580, Abcam, USA) and mouse anti-cardiac troponin T (1:100, ab8295, Abcam, USA), diluted using CytoVista's Antibody Dilution Buffer (V11305), were then added and allowed to bind for 2 days at 4°C on a shaker. Following three additional washes with the washing buffer, the organoids were incubated for one day at 4°C in a solution containing goat anti-rabbit Alexa Fluor 488 secondary antibody (1:200, A32731, Invitrogen, USA) and rabbit anti-mouse Alexa Fluor 647 secondary antibody (1:200, A56576, Invitrogen, USA) on a shaker. After three more washes, the organoids were transferred to DAPI staining solution (AC0065, ACMEC, China) and incubated for 4 hours on a 4°C shaker. After incubation, the organoids were washed three times. The specimens were cleared with a clearing Reagent (V11326, CytoVista, USA) prior to fluorescence imaging. Images were captured under a Leica confocal microscope, followed by statistical analysis using ImageJ software.
  2. **Purification and fluorescence identification of N_3_-exosomes.** Human umbilical cord mesenchymal stem cells (HMSCs) were cultured in Dulbecco's Modified Eagle's Medium/Ham's F12 nutrient medium (DMEM/F12, 1:1) supplemented with 10% fetal bovine serum and 1% penicillin/streptomycin at a temperature of 37°C within a humidified atmosphere containing 5% CO_2_. Exosomes derived from HMSCs were isolated from the culture medium using Invitrogen's Total Exosome Isolation Reagent, following the manufacturer's recommended protocol. For the generation of azide-modified exosomes (N_3_-exosomes), HMSCs were treated with 30μM N-azidoacetylglucosamine-tetraacylated (Glc) for a duration of 3 days, upon reaching 80% confluence. The purity of the isolated exosomes was determined using the BCA Protein Assay Kit (Beyotime, China).

To investigate Glc metabolism in HMSCs, the cells were cultured with Glc for 24 hours and then fixed using 4% paraformaldehyde after washing with PBS. Subsequently, the cells were incubated with FITC-DBCO (Ruixibio, China) to detect the presence of azide groups. Afterward, the cells were stained with TRITC-Phalloidin (Solarbio, China) to label F-actin within the HMSCs. To confirm azide modification on the exosomes, the purified exosomes were incubated with FITC-DBCO and Alexa Fluor® 647-conjugated anti-human CD63 Antibody (Biolegend, USA) for a duration of two hours at 37°C. Following this, the exosomes were subjected to PBS washes using an ultrafiltration tube with a 30 kDa cutoff (Millipore, Germany).

- 1. **Characterization of N_3_-exosomes.** The isolation of exosomes and N_3_-exosomes was accomplished using ultracentrifugation, following which they were prepared for nanoflow cytometry analysis and electron microscopy characterization. The purified particles were identified using FITC-conjugated Mouse Anti-Human CD63 (BD, USA) and FITC-DBCO, employing the N30E Flow NanoAnalyzer (Nanofcm, China). In this process, exosomes and N_3_-exosomes were respectively incubated with the CD63 antibody or DBCO-FITC for 30 minutes and 60 minutes. Subsequently, the exosomes underwent two PBS washes and a centrifugation step at 110,000×g for 70 minutes at 4°C. The purified exosomes and N_3_-exosomes were then resuspended in PBS for subsequent detection.

For transmission electron microscopy (TEM) analysis, a suspension of 10 µL of exosomes or N_3_-exosomes was carefully pipetted onto a carbon-coated copper grid, allowing the particles to settle for approximately 1 minute. Superficial liquid was then removed using a filter paper. Subsequently, 10 µL of uranyl acetate solution was added to the grid and allowed to settle for an additional 1 minute. Once again, the excess liquid was meticulously aspirated with a filter paper. The copper grid was left to air-dry at room temperature for several minutes. The dried grid was then placed under the Hitachi HT-7700 (Hitachi, Japan) electron microscope operating at an accelerating voltage of 100 kV for examination.

- 1. **Internalization of N_3_-Exosomes and** **DLC-VEGF Nanoparticles in H9C2 and HUVECs.** To validate the fusion of exosomes with H9C2 and HUVEC cells, N_3_-exosomes were labeled with FITC-DBCO. After labeling, the exosomes were incubated with H9C2 or HUVEC cells at 37℃ for 30, 40, 50, and 60 minutes. To visualize the internalization process, the cells were subsequently fixed with paraformaldehyde and stained with TRITC-Phalloidin (Solarbio, China). As an alternative approach, DLC-FITC nanoparticles, serving as a control for DLC-VEGF particles, were co-cultured with H9C2 cells or HUVECs. The internalization of the particles was documented by capturing fluorescent images using a fluorescence microscope (Zeiss Axio Abserver 7).

- 1. **Assessing endothelial cell migration with exosomes and DLC-VEGF nanoparticles.** The migration capacity of HUVECs treated with exosomes was evaluated through scratch assays utilizing the culture-insert two wells in μ-dish (Ibidi, Germany). Following the initial plating of HUVECs into each well and subjecting them to a 24-hour starvation period with EBM-2 basal medium (Lonza, Switzerland), the culture-insert well was removed to establish a 500 μm cell-free zone. The cells were then rinsed with PBS to remove debris. For the control group, HUVECs were kept in EBM-2 basal medium, whereas the experimental groups were treated with 100 μg/ml of exosomes or N_3_-exosomes. To track the wound area in the presence or absence of exosomes, microscope images were captured every 6 hours using an EVOS^®^FL Auto microscope (Thermo Scientific, USA). The area of the wound filled by migrating cells was analyzed using ImageJ software (NIH, USA), and the experiments were repeated at least three times independently. In a parallel manner, HUVECs were also tested for migration in the presence of DLC-VEGF nanoparticles at concentrations of 0.0 mM, 2.5 mM, and 5.0 mM.

- 1. **Evaluating endothelial cell tube formation with exosomes and DLC-VEGF nanoparticles.** Following a 24-hour starvation in EBM-2 basal medium, HUVECs were carefully washed with DPBS (Gibco, USA) to remove any debris. Cell suspensions were then seeded into wells of a 24-well plate that were pre-coated with growth factor-reduced Matrigel (BD, USA). The cells were incubated in EBM-2 basal medium supplemented with exosomes or N_3_-exosomes at specified concentrations (100 μg/ml) for a duration of 4 hours. For each concentration, three replicate wells were used to ensure a reliable statistical analysis. Tube formation was examined under a microscope (Olympus, Japan), and the length of the tubular structures, composed of capillary-like networks of more than six cells in length, was measured in each well and high-power field. The average tube length per well was calculated using ImageJ software. Furthermore, HUVECs were exposed to DLC-VEGF nanoparticles at concentrations of 0.0 mM, 2.5 mM, and 5.0 mM to assess their impact on tube formation.

- 1. **Preparation of egg shell microparticles (ESMPs).** The hard-boiled eggshell was meticulously detached from the egg, ensuring the integrity of the outer calcareous shell and its adherence to the inner proteinaceous membranes. The egg shell was then dice into fragments measuring less than 5 mm in diameter and desiccated thoroughly in an oven. To produce a consistent fine powder, the dried eggshell was preliminary chilled in liquid nitrogen for 10 minutes before being pulverized in a cryogenic grinder maintained at temperatures as low as minus 50℃. The resulting powder was then combined with deionized water to create an emulsion, which was passed through a filter assembly containing a 5 μm and a 2 μm microporous membrane under vacuum conditions. Post-filtration, the emulsified solution underwent freeze-drying before being placed in a desiccator for subsequent applications.

To functionalize the ESMPs, the filtered eggshell emulsion was subjected to a two-hour incubation with a 100 µM solution of dibenzocyclooctyne-N-hydroxysuccinimidyl ester (DBCO-NHS) (Macklin, China) at a temperature of 37℃. The resultant modified ESMPs, referred to as DBCO-ESMPs, were subsequently purified by centrifugation three times and then reconstituted in deionized water. In certain experiments, the reconstituted eggshell emulsion was incubated with a 100 μM N_3_-Cy3 (Ruixibio, China) solution at 37°C for two hours to evaluate the efficacy of the DBCO-NHS modification. Additionally, experiments were conducted where DBCO-ESMPs were co-incubated with N_3_-exosomes to examine the interaction between eggshells and exosomes.

- 1. **Tracking the Movement of ESMPs.** The motion trajectory of ESMPs were recorded using the time-lapse photography capabilities of the M230-M high-speed camera. Thereafter, Revealer Motion Analysis software (HF Agile Device Co., Ltd., China) was employed to process and analyze the captured footage. In the experiment designed to monitor the collective motion of numerous microparticles, a higher concentration of ESMP suspension was initially introduced into the micro-chamber. Subsequently, a micro syringe pump was used to gradually introduce citric acid solutions of different pH levels into the chamber, thereby observing the resulting particle motion. To track the trajectory of a single particle, a low concentration of ESMP suspension was injected into one chamber of a custom-made slide. As the citric acid solutions with varying pH values were allowed to flow from another chamber into the particle-containing chamber through a connecting channel, a high-speed camera captured and recorded the movement path of the microparticles.

- 1. **Preparation of vascular endothelial growth factor (VEGF)-****encapsulating** **dual complementary liposome (****DLC-VEGF).** The synthesis of DLC-VEGF nanoparticles utilized the extrusion method, as described in the reported literatures^[1]^. In essence, a lipid formulation composed of DOPC: DSPE-PEG-COOH (95:5, mol/mol) was employed to generate liposomes. A lipid mixture (50 μmol) was dissolved in chloroform and evaporated under a stream of dry nitrogen. The resulting lipid film was then dissolved in 1 ml of a DMSO:EtOH (7:3, v/v) mixture. The lipid solution was injected into 9 ml of a 240 mM sodium sulfate solution in PBS (pH 7.4) while being vigorously agitated to achieve a final lipid concentration of 5 mM. Following eight freeze-thaw cycles, a concentration of 2 μg/ml of VEGF was added and subjected to two additional freeze-thaw cycles. Subsequently, the lipid solution was extruded through a Northern Lipids Extruder using a 100-nm polycarbonate nanoporous membrane. The resulting liposomes were then washed with PBS (pH 7.4) at room temperature using an ultrafiltration tube with a 30 kDa cutoff (Millipore, Germany). The purified DLC-VEGF particles were mixed with 1-Ethyl-3-(3-dimethylaminopropyl) carbodiimide hydrochloride (EDC) and N-Hydroxysuccinimide (NHS) in PBS (pH 7.4) and incubated for 6 hours at room temperature. Any unreacted EDC and NHS were removed using an ultrafiltration tube with a 30 kDa cutoff. For cellular binding and internalization studies, a noncytotoxic FITC-dextran (molecular weight, 10 kDa)-containing liposome (DCL-FITC) was prepared as a substitute for the cytotoxic DCL-VEGF. The preparation process for DCL-FITC was similar to that of DCL-VEGF, except that 1 ml of lipid solution was combined with 9 ml of FITC-dextran solution (1 mg/ml).

- 1. **Determination of particle (N_3_-exosomes / ESMP / DLC-VEGF) size and zeta potential.** The particle size distribution and zeta potential of exosomes and N_3_-exosomes were characterized using the Nanobrook Omni nanoanalyzer (Brookhaven Instruments, USA). Each analysis was performed in triplicate, with the samples diluted 1/10, 1/100, and 1/1000 with PBS, respectively. A volume of 2 ml was employed as the sample size for each measurement after dilution. Concurrently, particle size and zeta potential assessments were also conducted on ESMPs and DLC-VEGF particles.

- 1. **Manufacturing effervescent microneedle (MN) patches.** The fabrication of MN patches was carried out utilizing polydimethylsiloxane (PDMS) micromolds, featuring a circular matrix with a diameter of 500 μm and a height of 1000 μm. The needle cavities were arranged in a 20 x 20 array with a 300μm bottom-to-bottom spacing.

To fabricate the MN patches, a two-step casting process was employed. The first casting solution consisted of 25% (w/w) solids, including PLGA in diglyme/deionized water (95%/5%, w/w). In certain experiments, a 0.1 mM N_3_-Cy3 solution was added to the first casting solution to generate Cy3-labeled MN patches. The MN mold was subjected to centrifugation at 3000 r/min using a spin coater (SETCAS Electronics Co., Ltd, China), and the first casting solution was applied to the top of the MN mold until it was filled. Following this, the MN mold was centrifuged at 8000 r/min for 15 minutes to eliminate air bubbles. Subsequently, the mold was placed in a 60°C oven under vacuum for 4 hours to dry the casting solution.

The second casting solution consisted of 13% (w/v) PVP in two molecular weights (360/55 kDa, 50/50%, w/w) and 4% (w/v) citric acid in pure ethanol, with 5% (w/v) sodium bicarbonate suspended within the solution. In certain experiments, a 0.1 mM N_3_-Cy5 solution (Shaanxi Xinyan Bomei Biotechnology Co., Ltd, China) was added to the second casting solution to generate Cy5-labeled MN patches. Two hundred microliters of the second casting solution were applied to the surface of the PDMS mold to form the effervescent patch base. For control groups with a noneffervescent base, the microneedle base was cast using the first casting solution. Following drying in a 40℃ drying oven for three days to ensure complete drying, the micromold was placed on ice for one minute and then carefully rolled down from both sides to obtain a complete MN patch. This patch was stored in a desiccator until it was ready for use.

- 1. **Preparation of DLC-VEGF-/Exosome-/ESMP-Exo-Loaded MN Patch.** Exosomes were concentrated to 60 mg/ml and quantified using a BCA Protein Assay Kit (Beyotime, China). These concentrated exosomes were then incubated with ESMPs (weight ratio of 1:30) at a temperature of 37℃ for 2 hours. Thereafter, 30 microliters of this mixture were added to the first casting solution, replacing the deionized water. In the group where exosomes were the sole loading, 30 μl of exosome concentrate was added directly. In parallel, an equivalent volume of eggshell microparticles was incorporated into the group intended for loading ESMPs only. Additionally, 10 mM DLC-VEGF nanoparticles were mixed into the second casting solution to prepare DLC-VEGF MN patches.

- 1. **Mechanical strength test.** The mechanical strength of MN patches was tested using the ElectroForce Biodynamic 5500 instrument (TA, USA). A 3×3 array, comprising 9 microneedles, was attached to a circular lower plate. The lower plate was pressed upward at a speed of 0.1 mm/s, whereby the upper plate exerted an axial force on the microneedles. Upon the failure of the microneedles, it is noteworthy that the applied force experienced an abrupt decrease. The maximum force exerted immediately prior to this drop was subsequently determined to be the failure force of the needles.

- 1. **Investigating the in vitro degradation of MN patch.** To investigate the degradation of MNs in vitro, the overall fabrication of MN patches was conducted using the first casting solution containing N_3_-Cy3. The MN patch was submerged in PBS and placed within a temperature-controlled shaker set at 37℃ and running at a speed of 100 rpm. At predetermined time intervals, samples of the PBS solution were collected, and fresh PBS solution was added to keep the volumes constant. The concentration of N_3_-Cy3 within the PBS solution was measured using a multifunctional microplate reader (Infinite^®^ M200 PRO, Tecan, Switzerland). The released percentage of N_3_-Cy3 was recorded at each time point, with the initial loading amount of N_3_-Cy3 in the MN patch serving as the control (100%). To evaluate the degradation of the effervescent patch base, it was fabricated using the second casting solution containing N_3_-Cy5, whereas the needle tips were cast with the first casting solution containing N_3_-Cy3. Adhering to the same experimental protocol, the MN patch was then immersed in PBS, and the concentration of N_3_-Cy5 within the PBS solution was measured at a specific time interval.

- 1. **MI Rat Model.** Sprague-Dawley (SD) rats (males, weighing between 250 to 300 g) were obtained from the Animal Center of the Army Medical University, China. All experimental protocols were stringently followed in accordance with the ethical guidelines for animal research, and were approved by the university’s Ethics Committee. The SD rats were induced into anesthesia through an intraperitoneal injection of pentobarbital sodium (30 mg/kg), and were intubated with cannulas connected to a rodent ventilator (ALC-V9, Shanghai Alcott Biotech Co., Ltd., China). Subsequently, a left thoracotomy was then performed at the third to fourth intercostal space to expose their hearts, and the left anterior descending (LAD) coronary artery was permanently ligated using a 6-0 nylon suture, resulting in an acute myocardial infarction (MI).

The MI rats were randomized into several treatment groups: (i) MI induction without any intervention (MI group), (ii) MI induction with a MN patch incorporating DLC-VEGF nanoparticles into the effervescent base (MI+DLC-VEGF group), (iii) MI induction with a MN patch containing exosomes within the needles (MI+Exosome group), (iv) MI induction with a MN patch containing ESMPs within the needles (MI+ESMP group), (v) MI induction with a MN patch that combined DLC-VEGF nanoparticles within the effervescent base and exosomes within the needles (MI+DLC-VEGF/Exo group), (vi) MI induction with a MN patch containing DLC-VEGF nanoparticles within the effervescent base and ESMP-exosome conjugates within the needles (MI+DLC-VEGF/ESMP-exo group), and (vii) normal hearts receiving MN patch application identical to group vi (Normal+DLC-VEGF/ESMP-exo group) as a control. Twenty-eight days post-MI, the rats were humanely euthanized to facilitate comprehensive analysis.

The MN patch was delivered into the myocardium using a negative pressure device. Specifically, the MN patch was placed within the vacuum filtration net cavity, with the needle tips pointing upwards. When suction was applied, the targeted myocardial area was drawn into the vacuum cavity at a negative pressure of approximately 0.08 kPa, thereby inserting the needle tips into the myocardium. Upon release of suction, the suction head was carefully retracted from the heart, allowing the MN patch to remain on the myocardial surface. A small amount of physiological saline was then applied to the surface of the MN patch, and the patch's base quickly dissolved.

- 1. **Echocardiography and Electrocardiogram Measurements.** To assess cardiac function in the rats, transthoracic echocardiography was performed at four specific time intervals: days 7, 14, 21, and 28 following the establishment of the MI model. The rats were sedated with 4% isoflurane and maintained under 2% isoflurane anesthesia. The precordial region was prepared, and a high-frequency ultrasound system (Vevo 2100, VisualSonics, Toronto, Canada) equipped with a linear array probe (MS400, operating at a frequency of 18-38 MHz) was utilized to capture images of the heart’s parasternal long-axis view in M-mode at the level of the papillary muscles. Parameters including ejection fraction, fractional shortening, left ventricular end-diastolic diameter, and left ventricular end-systolic diameter were computed from these images. Electrocardiogram (ECG) data were recorded 30 minutes post-ligation of the left anterior descending coronary artery to evaluate the impact of the MI. This ECG recording was carried out using a RM6240 multi-channel physiological signal acquisition and processing system (Chengdu Instrument Factory, China) with one- and two-lead detection.
  2. **Tetrazolium Chloride (TTC) Assay.** To differentiate between viable and non-viable cardiac tissue after MI, a TTC assay was performed. Following 30 minutes of LAD coronary artery occlusion, the heart was excised and gently washed with a physiological saline solution containing sodium heparin. Subsequently, the heart was immersed in a 2% solution of 2,3,5-triphenyltetrazolium chloride (Solarbio, China) and allowed to stain for 2 hours at a temperature of 37°C. After the staining process, the heart was fixed in a 4% formaldehyde solution overnight, with the fixation solution being changed once to remove any non-specific binding.

- 1. **Biosafety assessment.** Throughout both the experimental and control groups, all rats had blood samples drawn from the orbital venous plexus at specified time intervals using microhematocrit capillary tubes (Thermo Fisher Scientific, USA). The serum was then separated by centrifugation at 3000 r/min for 15 minutes at 4°C, and the resulting serum samples were stored at -80°C until they were ready for analysis. To evaluate liver and kidney function, ELISA kits (Jingmei Biotechnology, China) were used to quantify the serum levels of alanine aminotransferase (ALT), aspartate aminotransferase (AST), creatinine (CREA), and blood urea nitrogen (BUN). The assays were conducted in accordance with established protocols and the manufacturer's instructions. Furthermore, ELISA assays were conducted to assess changes in serum vascular endothelial growth factor (VEGF) levels.

- 1. **Heart morphometry studies**. Upon the completion of the four-week myocardial infarction induction period, all rats in the study were humanely euthanized after ultrasound data collection. The tissue samples were then fixed, embedded in paraffin, and cut into sections with a thickness of 4 μm, starting from the apex and extending to the level of the ligature. These sections were mounted on slides and stained with hematoxylin and eosin (HE), Masson's trichrome, and alizarin red. The staining was performed following standard protocols and the manufacturer's guidelines (Servicebio, China) to ensure reliability and consistency of the results. Images of each stained section were obtained using an automated pathological tissue digital scanning imaging system (Pannoramic MIDI, 3DHISTECH, Hungary).

- 1. **Immunofluorescence assay.** The immunofluorescence assay was conducted on paraffin-embedded heart sections to examine the expression of certain proteins critical for myocardial function and repair. For antigen retrieval, the sections were subjected to treatment with sodium citrate, followed by penetration with phosphate-buffered saline with Tween 20 (PBST) solution and blocking with 3% BSA solution. Overnight incubation at 4°C was carried out using the following primary antibodies: rabbit anti-Calponin 1 (1:100, ab46794, Abcam, USA), mouse anti-vWF (abx102082, Abbexa, Britain), mouse anti-α-SA (A7811, Sigma-Aldrich, USA), rabbit anti-Ki67 (ab15580, Abcam, USA), mouse anti-CD68 (ABM40050, Abbkine, China), rabbit anti-CD68 (ab283654, Abcam, USA), mouse anti-ARG1 (sc-271430, Santa Cruz, USA), rabbit anti-iNOS (ab178945, Abcam, USA), rabbit anti-TNF-α (ab205587, Abcam, USA), and rabbit anti-TGF-β (ab215715, Abcam, USA). The primary antibodies were then conjugated with FITC or Cy3 secondary antibodies (1:200; Abcam) and DAPI (Solarbio, China) was used for nuclear staining. High-resolution images were captured using the Pannoramic 250 Flash series digital scanner (3DHISTECH, Hungary), and tissue morphology was analyzed from these images using the NIH ImageJ software.
  2. **Quantitative determination of calcium ion.** The atrial and ventricular portions of the rat heart were sectioned horizontally, and these tissues were equally divided into five parts. The specimens were accurately weighed and added with homogenization medium (0.9% saline) in the ratio of weight (mg): volume (ul) = 1:9. Under ice water bath conditions, the mixture was mechanically homogenized and prepared into a 10% homogenate solution. The homogenate was then centrifuged at 2500 rpm for 10 minutes, and the supernatant was collected for the determination of calcium ion content. The measurement process was strictly carried out according to the operating instructions provided by the Nanjing Jiancheng Bioengineering Institute (China) for the Calcium Ion Assay Kit.

- 1. **Pig and rabbit thoracoscopic surgery.** Male minipigs (1 year old, 30–40 kg) were kept individually for one week. Before surgery, pigs were intramuscular injected with 15 mg/kg sodium pentobarbital solution (Shanghai YuanSiBiaoWu Technology Co., Ltd., China). After induction of anesthesia, the pig was fixed supine on the operating table and ventilated by endotracheal intubation and maintained with 2% isoflurane (RWD Life Science, China). Afterward, the pig was turned to the right lateral position. An incision was made between the pig's second and third ribs, through which the endoscope was delivered to the heart. Guided by the endoscope, a MN patch was inserted into the pig's ventricular wall using a custom-designed vacuum device suction device through the opening between the third and fourth ribs. Subsequently, a minimal amount of normal saline was applied to the MN base to expedite its degradation. The entire procedure was executed using the RT-600 endoscopic image processing system (Hangzhou Kangyou Medical Equipment Co., Ltd, China) for comprehensive image acquisition and processing. The experimental demonstration on rabbit hearts was similar. In the experiment, New Zealand white rabbits were anesthetized with 30 mg/kg sodium pentobarbital solution.

**References**

[1] a)P. Guo, J. Yang, D. R. Bielenberg, D. Dillon, D. Zurakowski, M. A. Moses, D. T. Auguste, *J Control Release* **2017**, 263, 57; b)P. Guo, J. Yang, D. Liu, L. Huang, G. Fell, J. Huang, M. A. Moses, D. T. Auguste, *Sci Adv* **2019**, 5, eaav5010.

**2. Supplementary Figures**


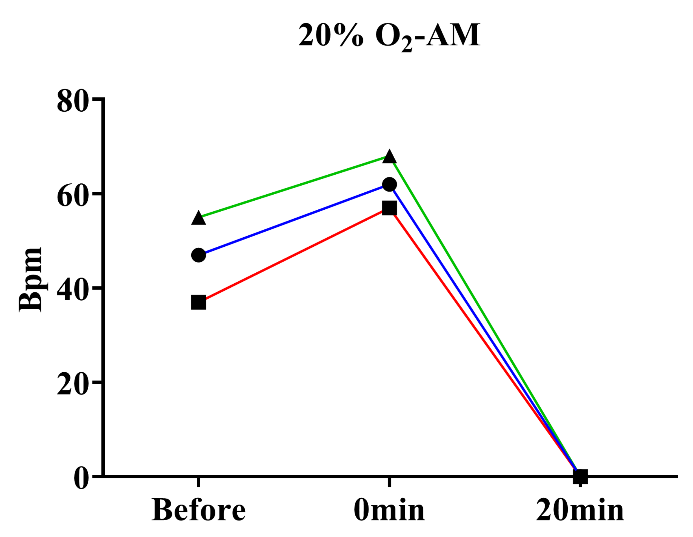


**Fig.S1.** **Monitoring the beating frequency of HCOs after 20 minutes of acidic incubation in an environment with 20% oxygen.**


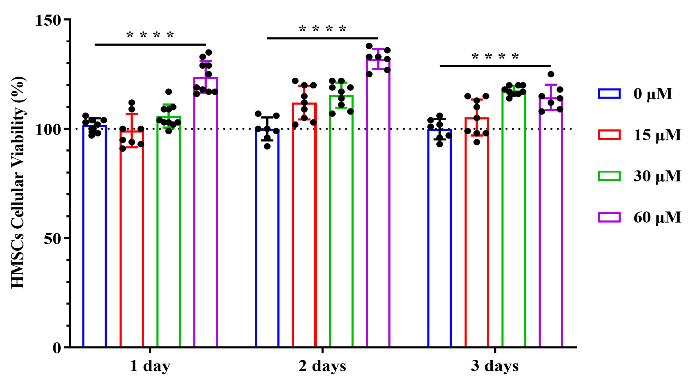


**Fig.S2. Assessing the viability of HMSCs treated with Glc**. HMSCs were cultured with varying concentrations of Glc for 24 hours, 48 hours, and 72 hours, respectively. The cell viability was assessed using the CCK-8 assay. n≥7 per group. All data were presented as mean ± SD. Comparisons among groups were performed using one-way ANOVA, and statistical significance was indicated by the asterisks (*) above the lines. ****P < 0.0001.


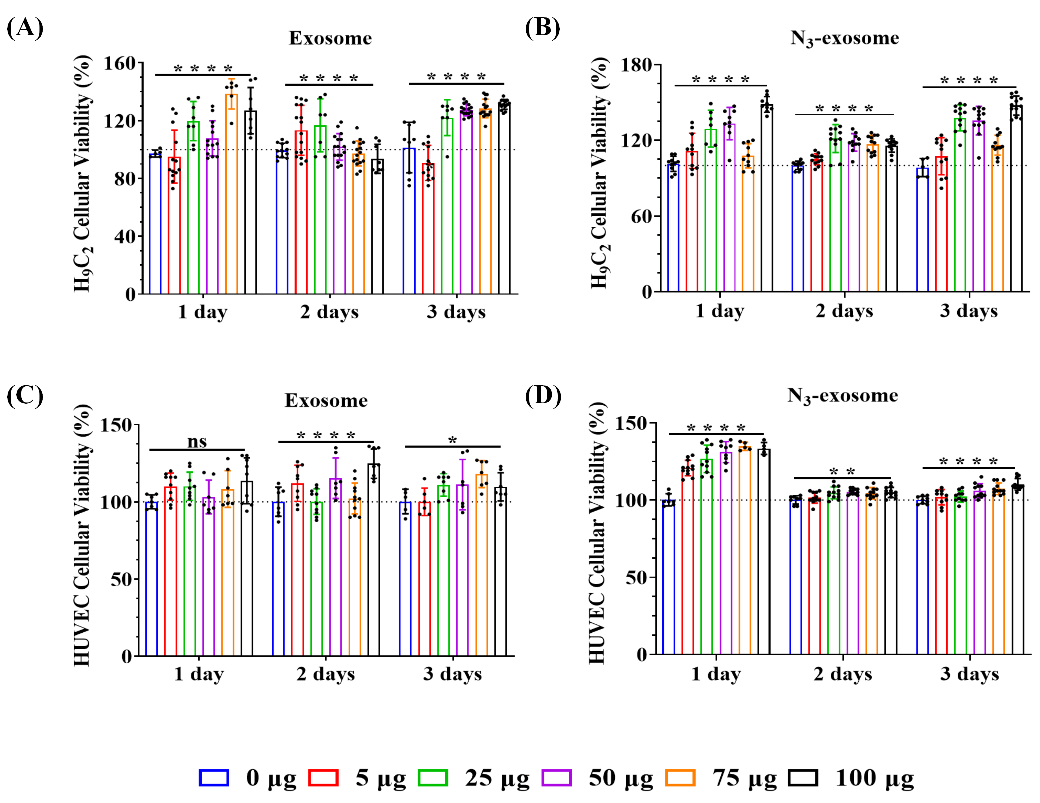


**Fig.S3. Evaluating the metabolism of N_3_-exosomes in cells**. (**A-D**) H9C2 or HUVECs were incubated with designated concentrations of exosomes for 24 hours, 48 hours, and 72 hours, respectively. Cell viability was determined using the CCK8 assay (n≥5). All data were presented as means ± SD. Comparisons among groups were performed using one-way ANOVA, and statistical significance was indicated by the asterisks (*) above the lines. NS indicates P > 0.1234. **P < 0.0021 and ****P < 0.0001.


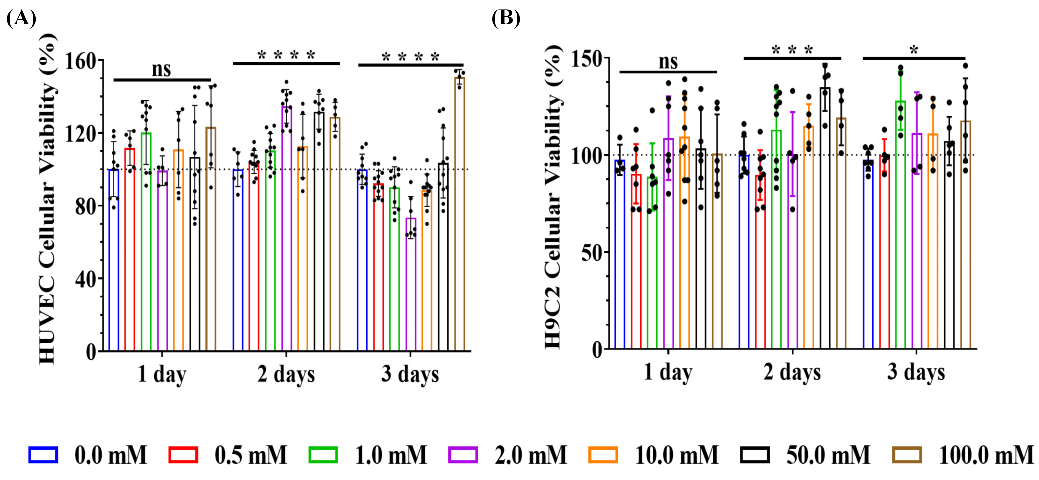


**Fig.S4. Assessing the metabolism of DLC-VEGF in cells.** (**A-B**) CCK8 assays were performed to analyze the metabolism of DLC-VEGF in H9C2 and HUVECs (n≥5). All data were presented as means ± SD. Comparisons among groups were performed using one-way ANOVA, and statistical significance was indicated by the asterisks (*) above the lines. NS indicates P > 0.1234. *P < 0.0332, ***P < 0.0002, and ****P < 0.0001.


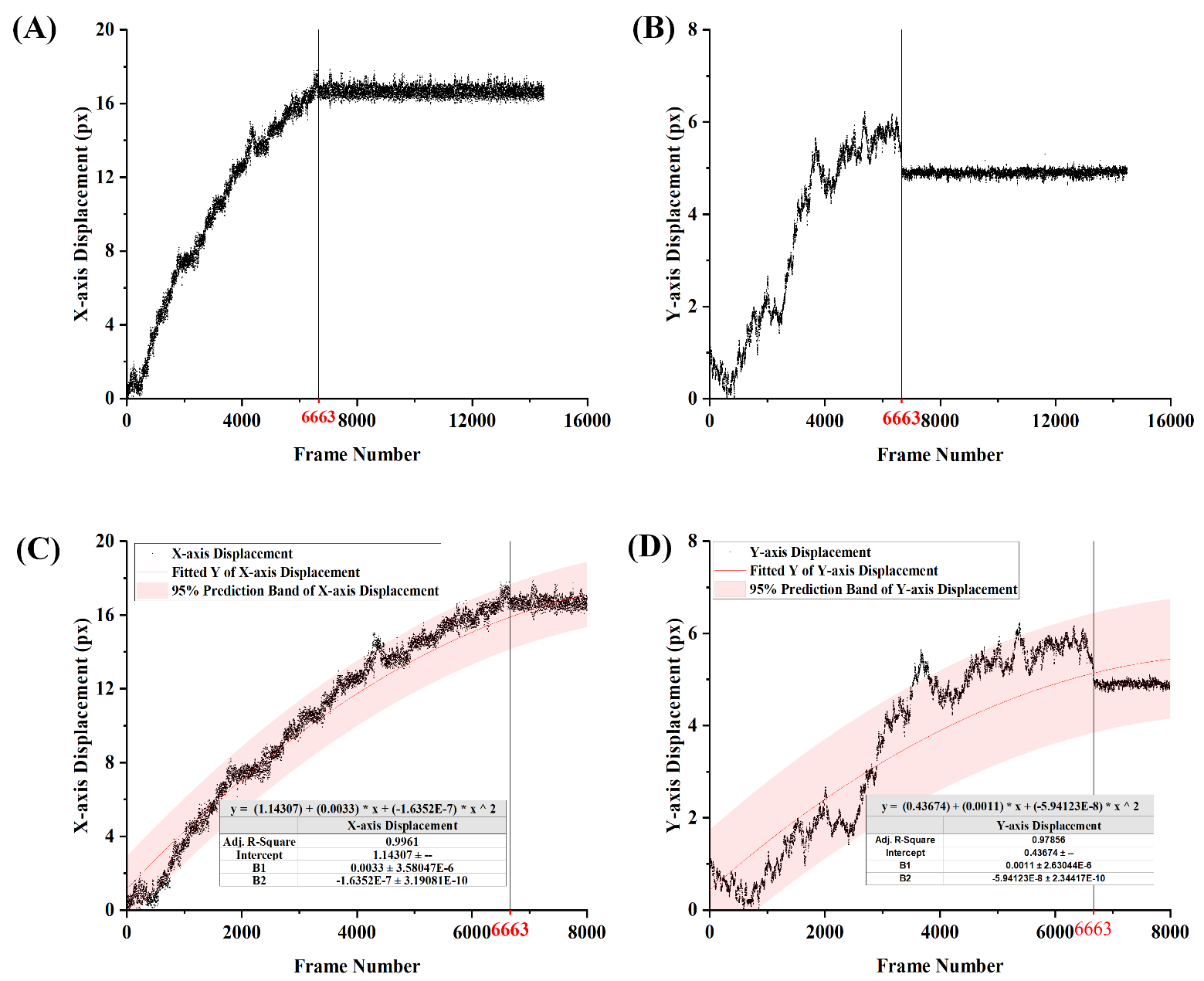


**Fig.S5. Investigating the motion of an ESMP under citric acid solution at pH=2.** (**A-B**) Analyzing the particle movement in both the X and Y directions, indicating a pause at the 6,663rd frame. (**C-D**) Analyzing the motion trajectory of images captured from 0 to 8,000 frames.


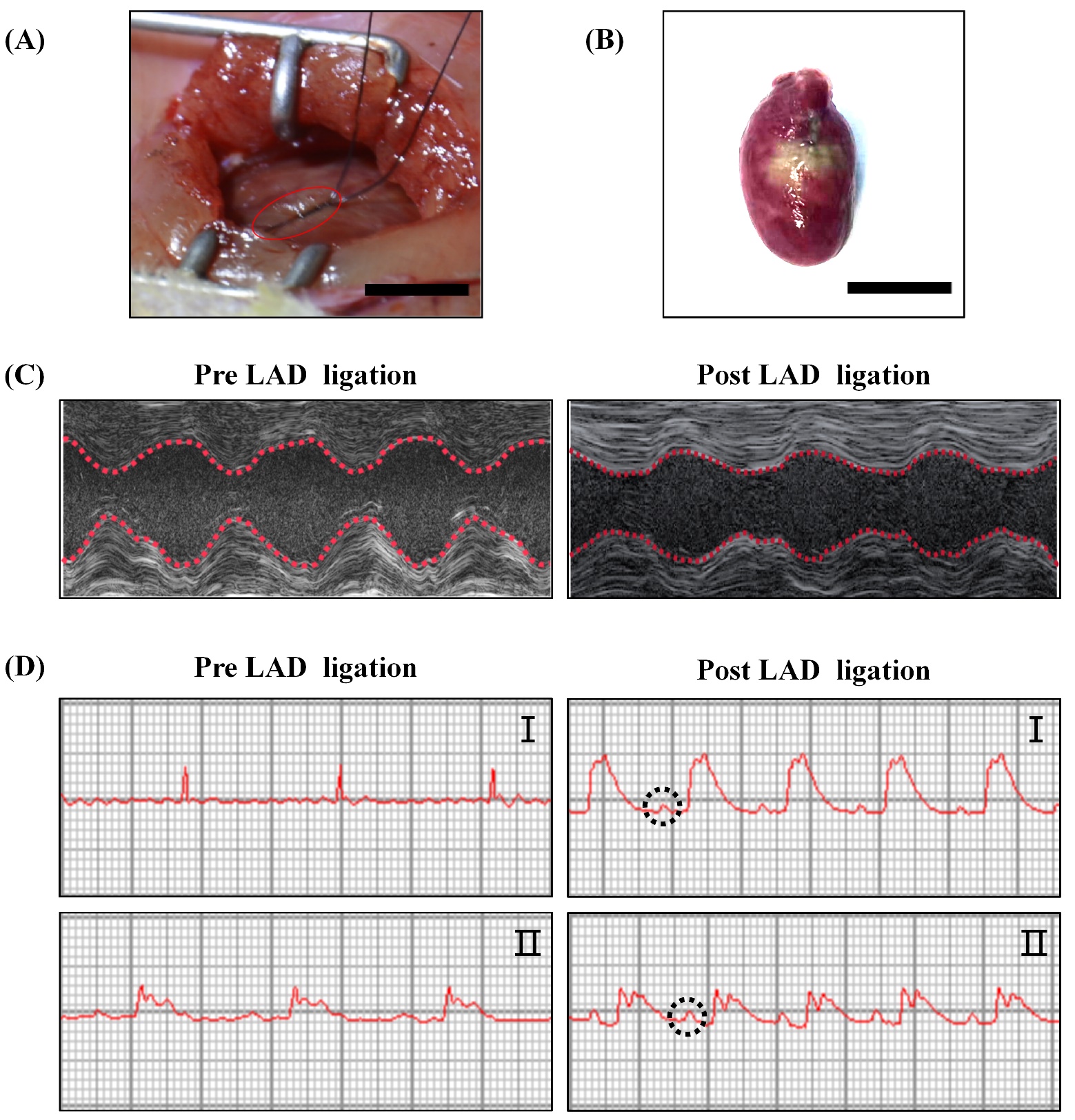


**Fig.S6. Establishing myocardial infarction in rats through left anterior descending (LAD) artery occlusion.** (**A**) Digital image illustrating LAD occlusion in a rat. Scale bar, 1cm. (**B**) TTC staining macroscopic images showing the infarcted region of a rat heart. Scale bar, 5mm. (**C-D**) Changes in ultrasound and electrocardiographic (ECG) parameters from pre-LAD occlusion to post-LAD occlusion in rat studies. The decrease in pulsation amplitude of the left ventricular anterior wall and the elevation of the ST segment after LAD occlusion confirmed the successful induction of MI.


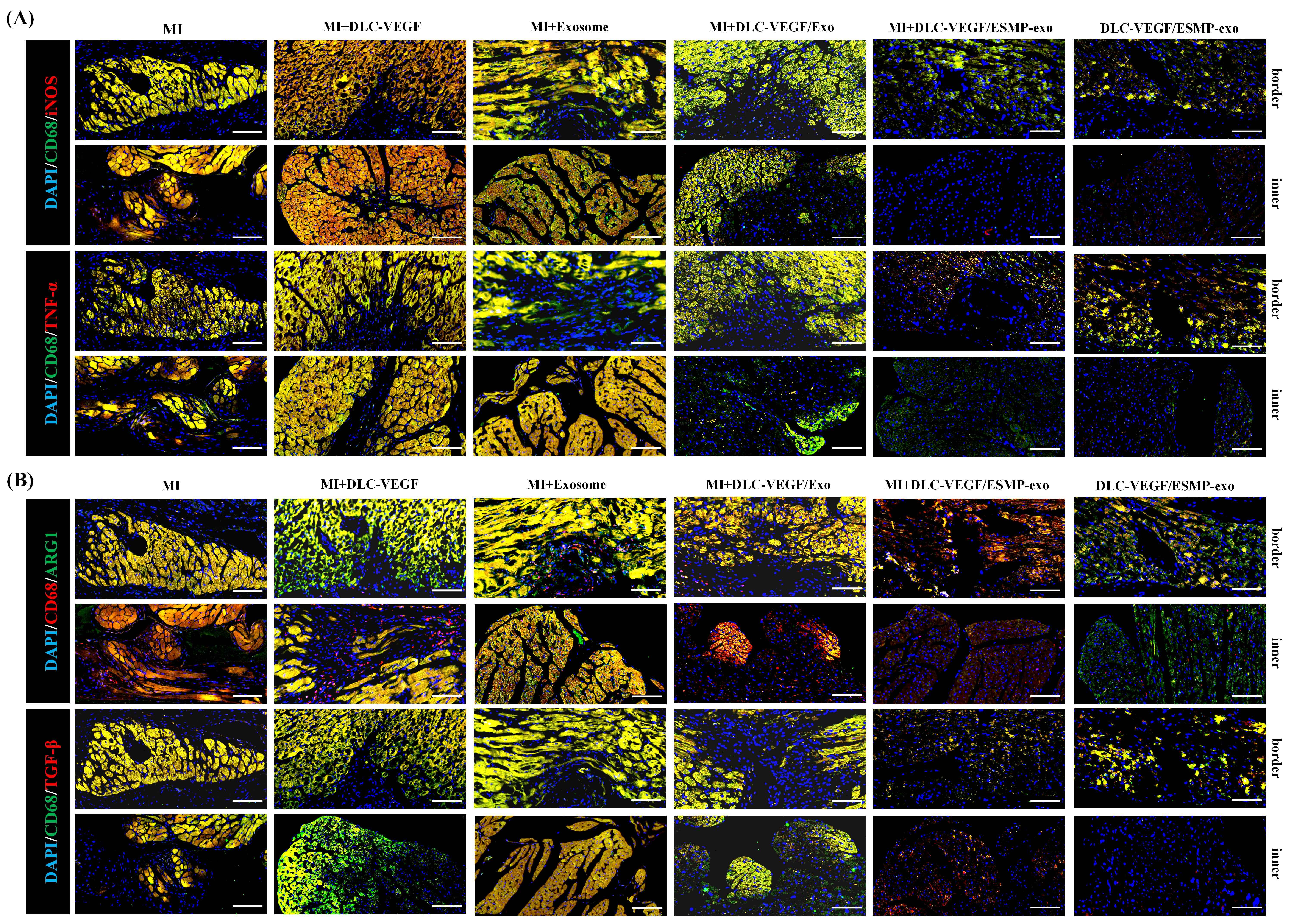


**Fig.S7. Analyzing inflammatory cell infiltration.** A study was conducted 28 days after MI to evaluate the infiltration of macrophages in rat heart tissues via immunofluorescence analysis. (**A**) Detection of M1-type macrophages: Anti-CD68 (green) was co-localized with either anti-iNOS (red) or anti-TNF-α (red) to identify the pro-inflammatory response within the border and inner regions of the infarcted area. Scale bar: 100 μm. (**B**) Identification of M2-type macrophages: Anti-CD68 (red) was combined with anti-Arg1 (green), or anti-CD68 (green) paired with anti-TGF-β (red), to identify the anti-inflammatory response in the border and inner regions of the infarcted area. Scale bar: 100 μm.


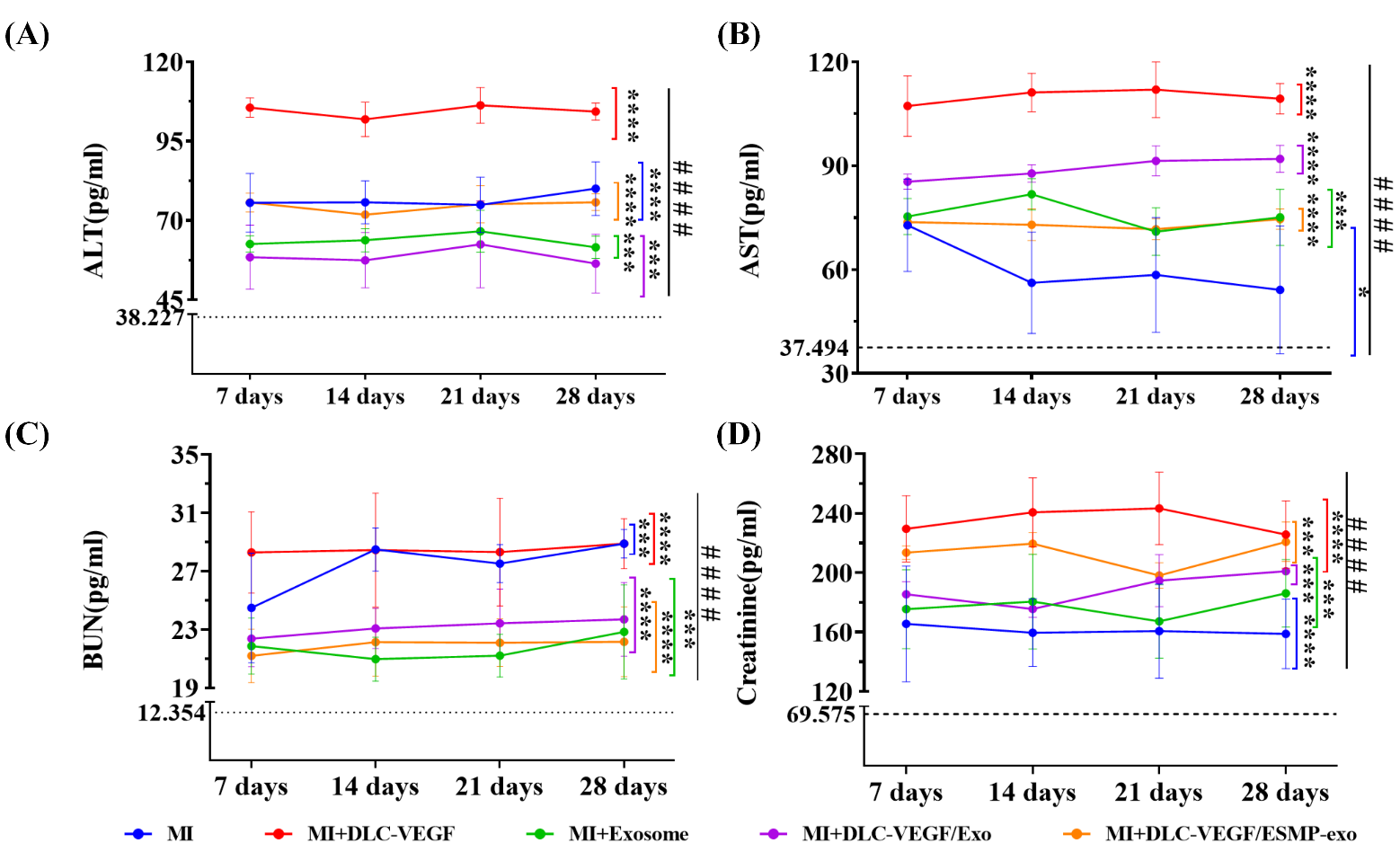


**Fig.S8. Evaluating liver and kidney function in rats.** (**A-B**) Assessing the potential hepatotoxicity of MN patches via ALT and AST concentrations. (**C-D**) Evaluating the potential kidney toxicity using blood urea nitrogen BUN and creatinine concentrations. n = 3 in each group. The black dotted line represented the normal value of rats (average of 4 rats). All data were presented as mean ± SD. Comparisons among groups were conducted using one-way ANOVA, and statistical significance was denoted by the hashtag (^#^) above the line. Comparisons between samples were performed by one-sample t test, and statistical significance was indicated by asterisks (*) above the lines. ***P < 0.0002, and ****^(####)^ P < 0.0001.


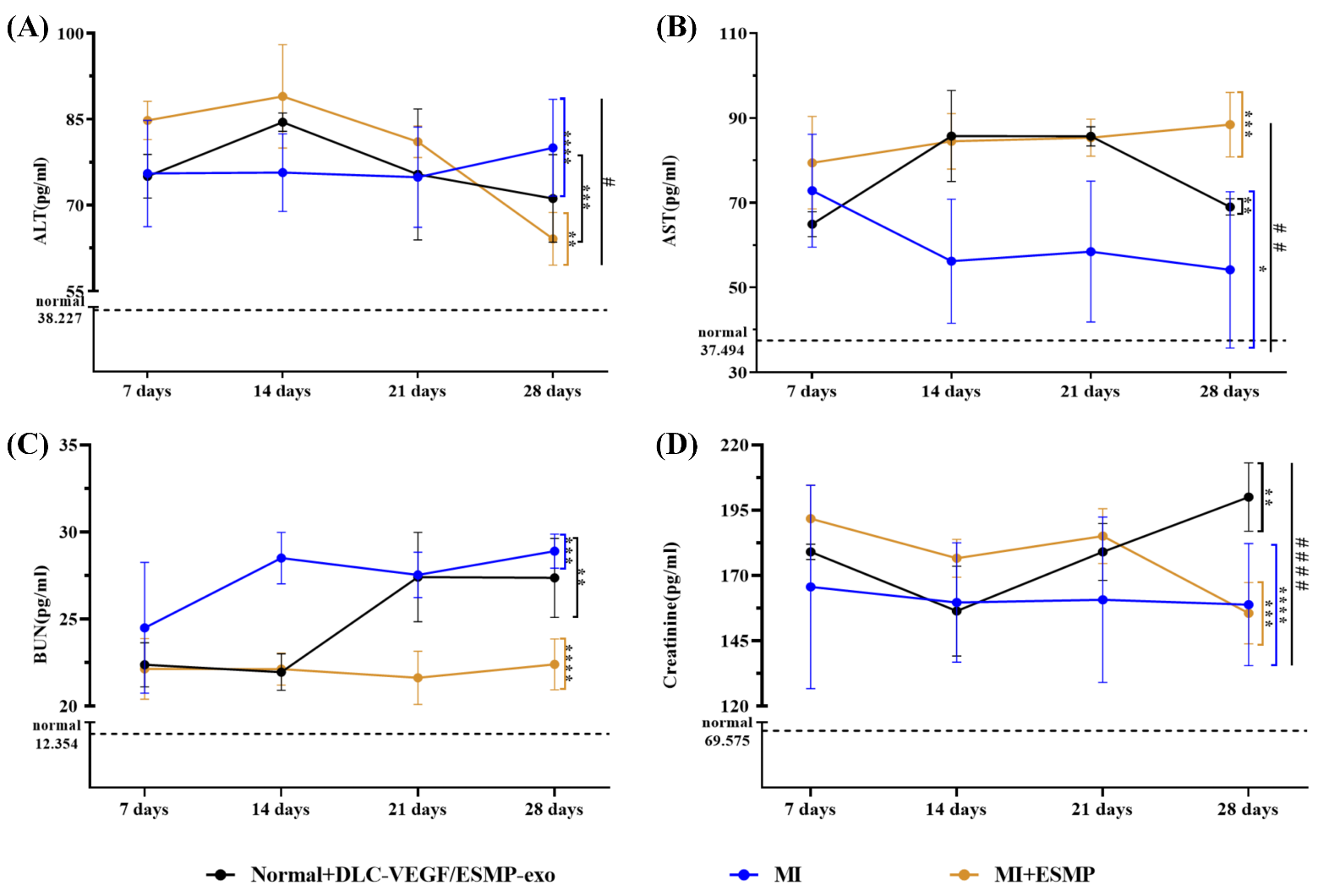


**Fig.S9. Assessing the potential metabolic toxicity of ESMPs in rats.** (**A-D**) Testing the concentrations of ALT, AST, creatinine, and BUN to determine the potential impact on liver and kidney function in rats. n = 3 in each group. The black dotted line represented the normal value of rats (average of 4 rats). All data were mean ± SD. Comparisons among groups were performed using one-way ANOVA, and statistical significance was indicated by the hashtag (^#^) above the line. Comparisons between samples were performed by one sample t test, and statistical significance was indicated by asterisks (*) above the lines. NS indicates P > 0.1234. *^(#)^ P < 0.0332, and **^(##)^ P < 0.0021. ***P < 0.0002, and ****^(####)^ P < 0.0001.


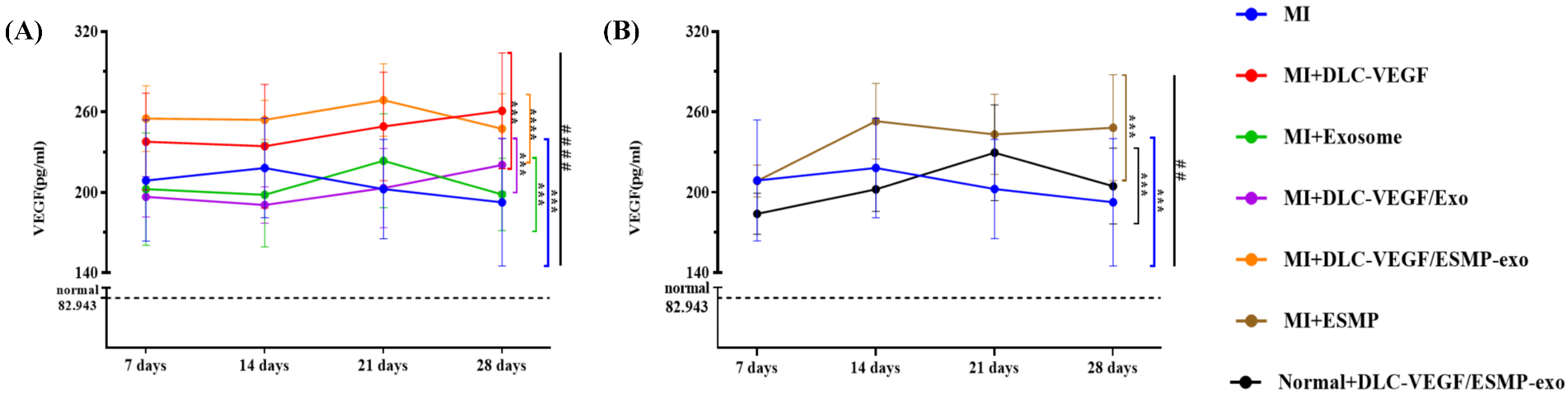


**Fig. S10. Analyzing VEGF Levels in rat serum.** (**A-B**) VEGF changes in different groups detected by ELISA during the 28-day experiment. The black dotted line represented the normal values of rats (average of 4 rats). n = 3 in each group. All data are mean ± SD. Comparisons among groups were performed using one-way ANOVA, and statistical significance was indicated by the hashtag (^#^) above the line. Comparisons between samples were performed by one sample t test, and statistical significance was indicated by asterisks (*) above the lines. ^##^P < 0.0021. ***P < 0.0002, and ****^(####)^ P < 0.0001.


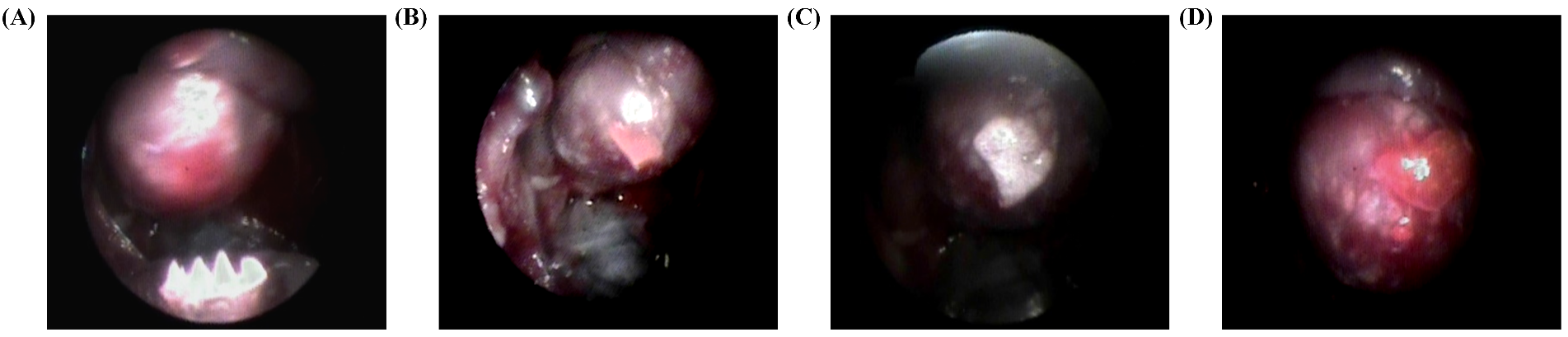


**Fig. S11. Video-assisted thoracoscopic surgery for MN patch application on rabbit hearts.** (**A-B**) Delivering the MN patch into the rabbit heart using a negative pressure device. (**C**) The effervescent base rapidly degrading and generating a substantial number of bubbles. (**D**) The remaining MN patch firmly attached to the heart surface. Images from **Video S11**.

**3. Supplementary Movies**

**Video S1. HCOs were placed in maintenance medium for a one-hour incubation under hypoxic conditions.**

**Video S2. HCOs were placed in acidic medium for a one-hour incubation under hypoxic conditions.**

**Video S3. HCOs were placed in acidic medium for a one-hour incubation under hypoxic conditions, while combined application of ESMP+Exo.**

**Video S4. ESMPs driven by 1 M citric acid solution in a microfluidic chamber.**

**Video S5. ESMPs driven by a citric acid solution at pH = 2.0 in a microfluidic chamber.**

**Video S6. ESMPs driven by a citric acid solution at pH = 5.0 in a microfluidic chamber.**

**Video S7. Movement of an ESMP larger than 100 μm in diameter driven by 1 M citric acid solution.**

**Video S8. Movement of an ESMP about 1 μm in diameter driven by ddH_2_O.**

**Video S9. An ESMP with a diameter of about 1 μm driven by a citric acid solution at pH = 2.0.**

**Video S10. The effervescent base rapidly degrading in vivo, generating a substantial number of bubbles.**

**Video S11. A Comprehensive process of the application of a MN patch on the rabbit heart via thoracoscopic surgery.**

**Video S12. A Comprehensive process of the application of a MN patch on the pig heart via thoracoscopic surgery.**
